# Machine learning approaches for phishing detection and cybersecurity awareness in digital infrastructure

**DOI:** 10.3389/frai.2026.1875321

**Published:** 2026-10-05

**Authors:** Parves Alam, Md Azizul Rahaman, Jubayer Uddin Shamim, Shakhawat Hossain Refat, Wali Ahmed, Pappu Roy, Shaon Reza, Samuel Kim

**Affiliations:** 1 Graduate School of Technology (GST), Touro University, New York, NY, United States; 2 Department of Computer Science, University of Bridgeport, Bridgeport, CT, United States; 3 Department of Computer Science, University Geomatika Malaysia, Kuala Lumpur, Malaysia; 4 Department of Computer Science, Stanford University, Stanford, CA, United States

**Keywords:** CNN-BiLSTM, cybersecurity awareness, explainable artificial intelligence, gradient boosting, machine learning, phishing detection, SHAP, Wilcoxon signed-rank test

## Abstract

**Introduction:**

Phishing remains a dominant socio-technical threat to digital infrastructure, serving as an entry vector for credential-theft, ransomware, and supply-chain incidents. The machine learning literature on phishing-URL detection remains fragmented across heterogeneous datasets, inconsistent leakage controls, and explanation pipelines seldom cross-validated by independent attribution methods.

**Methods:**

An end-to-end, leakage-controlled machine learning framework was developed over a balanced corpus of 11,430 instances and 87 engineered URL-lexical, HTML-content, and external-reputation features, with all preprocessing transforms fit on the training partition only. Eleven heterogeneous classifiers were benchmarked; the best classical model (LightGBM) was repurposed as a deterministic feature extractor whose out-of-fold posterior probabilities augmented the input to a hybrid CNN-BiLSTM. Statistical significance was assessed via Wilcoxon signed-rank tests with Holm-Bonferroni correction on 10-fold cross-validation *F*_1_ scores, and two explainable-AI methods (Tree SHAP and Permutation Importance) were applied alongside ablation studies.

**Results:**

LightGBM attained *F*_1_ = 0.9699 and AUC = 0.9943, significantly outperforming every other classifier (*p* ≤ 0.002) except XGBoost (*p* = 0.375). The CNN-BiLSTM hybrid attained *F*_1_ = 0.9652 and recall = 0.9703. SHAP and Permutation Importance agreed on 11 of 15 top-ranked features, separating external-reputation indicators (Google indexing, page rank, domain age, web traffic) from URL-lexical complexity indicators (length, dots, hyphens, phishing hints).

**Discussion:**

Strict leakage control confirms that the attainable performance is intrinsically high rather than artifactual. The convergent dual-method explanations translate into six candidate cybersecurity-awareness guidelines for end-user training, connecting technical detection to the human factors that shape enterprise security posture.

## 1 Introduction

The democratization of online services, the migration of critical infrastructure to cloud-native architectures, and the post-pandemic normalization of remote work have collectively transformed the attack surface of contemporary digital infrastructure (Anti-Phishing Working Group, 2023). Within this expanded surface, phishing has consolidated its position as the dominant threat vector. The Anti-Phishing Working Group reports that the volume of unique phishing sites observed quarterly has roughly tripled over the past 5 years, and the Verizon Data Breach Investigations Report identifies phishing as the initial-access mechanism for a majority of confirmed credential-theft and ransomware incidents (Verizon, 2023). Although phishing is fundamentally a social-engineering attack that exploits the trust heuristics of human users, the technical artifact through which the attack is delivered, namely the URL, is sufficiently rich in distributional structure to enable automated detection through machine learning (Aljofey et al., 2022).

The literature on machine learning-based phishing detection now spans more than a decade. Early approaches relied on hand-crafted blacklists and rule-based heuristics over URL strings, which suffered from poor generalization to unseen domains and required continual manual curation (Prakash et al., 2010). The introduction of supervised learning over engineered features, including URL length, special-character counts, sub-domain depth, and the presence of brand-mimicking tokens, substantially improved coverage and is the methodological tradition into which the present study falls (Mohammad et al., 2014; Şahingoz et al., 2019). More recently, deep-learning approaches that ingest raw URL strings via convolutional and recurrent encoders have shown competitive performance, particularly when trained on very large corpora (Le et al., 2018; Tajaddodianfar et al., 2020). Hybrid models that fuse engineered features with learned representations occupy a middle ground and have been reported to combine the data efficiency of the former with the representational flexibility of the latter (Ozcan et al., 2023).

Despite this maturity, three methodological problems recur with sufficient frequency to warrant systematic attention. First, the leakage problem: a non-trivial fraction of published pipelines fit feature-selection or scaling transforms on the union of training and test data, which inflates reported metrics and confounds comparative claims (Kaufman et al., 2012). Second, the significance problem: model rankings are typically reported on point estimates of accuracy or *F*_1_-score, with little attention to whether the observed gaps survive the noise of cross-validation. The Wilcoxon signed-rank test is the appropriate non-parametric instrument for this purpose (Demšar, 2006), but its use remains uneven in the cybersecurity machine learning literature. Third, the explainability problem: while SHAP (Lundberg and Lee, 2017) and permutation-based attributions (Breiman, 2001) are now standard tools, single-method explanations are vulnerable to method-specific artifacts, and convergent attributions across orthogonal frameworks are seldom reported.

The present study is motivated by the conjunction of these three concerns and by an additional, application-oriented observation: The machine learning literature on phishing rarely closes the loop between predictive findings and the actionable cybersecurity-awareness recommendations that constitute the strongest practical defense against phishing. End-user susceptibility to phishing is repeatedly shown to be the binding constraint on enterprise security posture (Jampen et al., 2020), and any technical detection pipeline that does not surface its findings in a form pedagogically useful for training programmes leaves a substantial fraction of its potential value unrealised.

Against this background, the contributions of this study are 4-fold. First, the study develops and empirically validates a complete, leakage-controlled machine learning pipeline for phishing-URL detection over a balanced corpus of 11,430 URLs and 87 engineered features. Second, it benchmarks 11 heterogeneous classifiers spanning linear, tree-based, ensemble, instance-based, probabilistic, and shallow-neural-network paradigms and verifies the statistical significance of the resulting performance gradient through Wilcoxon signed-rank tests on 10-fold cross-validation *F*_1_ scores. Third, it proposes a hybrid CNN–BiLSTM architecture in which the best-performing classical model serves as a deterministic feature extractor, supplying posterior-probability augmentations to the deep network, and demonstrates that this hybrid attains a recall-favorable operating point of practical security value. Fourth, it conducts dual-method explainable-AI analysis using Tree SHAP and Permutation Importance, documents strong inter-method agreement, and translates the convergent explanations into a compact set of cybersecurity-awareness rules suitable for end-user training. A series of ablation studies isolates the marginal contribution of each major design choice. The remainder of the study is structured as follows: Section 2 surveys related work; Section 3 details the proposed methodology; Section 4 reports the experimental results, including ablation analyses; Section 5 discusses the implications for cybersecurity awareness; and Section 6 concludes.

We state the scope of the contribution precisely to avoid overclaiming. None of the individual ingredients, leakage-controlled preprocessing, Wilcoxon-based comparison, posterior augmentation, CNN–BiLSTM hybridization, or post-hoc explainability, is in isolation novel to phishing detection. The contribution is instead methodological and integrative: The value lies in combining strict leakage control with non-parametric significance testing and family-wise error correction, in cross-validating the explanation through two orthogonal attribution methods rather than relying on a single method, and in carrying the resulting convergent feature hierarchy through to an explicit, falsifiable set of end-user awareness guidelines. We are explicit that the CNN–BiLSTM hybrid does not improve aggregate *F*_1_ over the strongest single classifier; its role is to demonstrate a leakage-safe mechanism for injecting tree-ensemble knowledge into a deep model and to expose a recall-favorable operating point, and we evaluate it on those terms rather than on aggregate accuracy.

## 2 Related work

### 2.1 Engineered-feature approaches to phishing detection

The earliest generation of machine learning phishing detectors relied on rule-based and decision-tree models over a small number of URL-lexical features (Garera et al., 2007). Mohammad et al. (2014) extended this paradigm by curating a thirty-feature set drawn from URL syntax, page content, and WHOIS metadata and demonstrated that an ensemble of neural networks could exceed 92% accuracy on a balanced corpus. Şahingoz et al. (2019) explored a broader feature universe and reported that random-forest classifiers consistently outperformed linear and instance-based alternatives. The dataset used in the present study extends these traditions by combining URL-lexical descriptors with HTML-content descriptors and external-reputation indicators including page rank, Google indexing, domain age, and web traffic (Hannousse and Yahiouche, 2021). The empirical regularity that emerges from this body of work is consistent: Tree-based ensembles, including random forests, extra trees, and gradient boosting, tend to dominate other classical model families on engineered phishing features, in part because they accommodate the heavy-tailed and discontinuous distributions characteristic of URL-lexical data without requiring scaling or transformation.

### 2.2 Gradient boosting in cybersecurity

The XGBoost framework (Chen and Guestrin, 2016) and the subsequent LightGBM framework (Ke et al., 2017) have become standard tools for tabular-data classification in cybersecurity applications, including malware detection, intrusion detection, and credit-card fraud detection. Their dominance reflects three properties: histogram-based split-finding that scales to large feature counts, regularized leaf-weight learning that controls overfitting, and native handling of missing values. In the phishing-detection setting, several studies have reported that LightGBM and XGBoost achieve test *F*_1_-scores in the 0.96–0.98 range on engineered-feature datasets of the size used here (Alsariera et al., 2020; Gandotra and Gupta, 2021). The present study confirms this regularity and additionally demonstrates that the gap between LightGBM and XGBoost is not statistically significant on the corpus considered, an observation that has practical consequence for deployment decisions where inference latency or model size may differ between the two frameworks.

### 2.3 Deep learning over URLs

Parallel to the engineered-feature literature, a strand of work treats URLs as raw character sequences and applies convolutional and recurrent networks directly. Le et al. (2018) introduced URLNet, a character-level convolutional network for URL classification, and Huang et al. (2019) extended this approach with attention mechanisms. Ozcan et al. (2023) developed a hybrid that combines engineered features with a CNN-LSTM character-level pathway. The CNN–BiLSTM architecture proposed here differs in two respects. First, it operates on engineered features augmented with the posterior probabilities of a high-performing tree ensemble, rather than on raw URL characters; this design preserves the interpretability of the underlying features while still allowing the deep network to discover non-linear interactions among them. Second, the parallel CNN and BiLSTM branches are merged via global average pooling and concatenation rather than sequential composition, reducing parameter count and improving training stability on the relatively modest dataset size considered. Bidirectional LSTMs in particular have been shown to capture context-sensitive dependencies that unidirectional recurrences miss (Schuster and Paliwal, 1997; Graves and Schmidhuber, 2005), and this advantage carries over to the engineered-feature setting where feature ordering can be exploited by the convolutional branch.

### 2.4 Explainable artificial intelligence in phishing detection

The use of explainable-AI techniques to interpret phishing classifiers has grown rapidly. Lundberg and Lee (2017) introduced SHAP, which provides theoretically grounded local and global feature attributions through Shapley values from cooperative game theory. Lundberg et al. (2020) subsequently developed Tree SHAP, an exact polynomial-time algorithm for tree ensembles. Permutation Importance (Breiman, 2001) provides an orthogonal, model-agnostic alternative whose attributions are derived from observed performance degradation under feature perturbation. Although both methods are now common in the phishing literature, they are typically reported in isolation, leaving open the question of whether their attributions are mutually consistent. The present study addresses this gap by applying both methods to the same model and quantifying the overlap between their top-ranked features.

### 2.5 Cybersecurity awareness as a defensive layer

Cybersecurity awareness research (Jampen et al., 2020; Parsons et al., 2017) consistently finds that the human user is the binding constraint on phishing defense: Even highly accurate technical detectors will be circumvented by some fraction of attacks that reach end users, and the susceptibility of those users to social-engineering cues determines the residual risk. The most effective awareness programmes translate technical insights into a small number of memorable rules that users can apply during everyday browsing (Abawajy, 2014). The convergent explanations produced by SHAP and Permutation Importance in this study lend themselves naturally to such translation, and Section 5 develops this connection.

Table 1 situates the present study against representative prior systems along four axes that bear directly on the comparability of reported results: the dataset and its scale, whether explicit leakage controls were reported, the statistical treatment of model comparison, and the explainability approach. The table makes clear that while individual elements of the present pipeline have precedent, their combination under explicit leakage control with non-parametric significance testing and dual-method explanation is not standard in the prior literature, which provides the appropriate context for interpreting the *F*_1_ = 0.9699 reported here.

**Table 1:** Comparison with representative prior phishing-detection systems.

Study	Dataset (size)	Leakage control	Significance test	XAI	Reported *F*_1_/Acc.
Mohammad et al. (2014)	Custom (1,400)	Not reported	None	None	0.92 (Acc.)
Şahingoz et al. (2019)	Custom (73,575)	Not reported	None	None	0.98 (Acc.)
Alsariera et al. (2020)	UCI (11,055)	Not reported	None	None	0.97 (Acc.)
Hannousse and Yahiouche (2021)	This corpus (11,430)	Not reported	None	None	0.966 (Acc.)
Ozcan et al. (2023)	Ebbu2017 & PhishTank	Not reported	None	None	0.98 (Acc.)
**This work**	This corpus (11,430)	**Yes**	**Wilcoxon**	**SHAP + Perm**.	**0.9699 (*F*_1_)**

“Leakage control” denotes whether preprocessing transforms were reported as fit on training data only; “Significance” denotes the statistical treatment of model comparison; “XAI” denotes the explainability method. Reported metrics are quoted as published in the original studies and are not directly comparable across differing datasets and protocols. Bold values indicate the best-performing entry for the corresponding column metric.

## 3 Methodology

### 3.1 Dataset and preliminary inspection

The empirical investigation was conducted on a publicly available, large-scale phishing URL corpus comprising *N* = 11, 430 instances and *d* = 89 attributes, of which 87 are engineered numerical features, one is the raw URL string, and one is the categorical target (Hannousse and Yahiouche, 2021). The class distribution is perfectly balanced with 5,715 legitimate and 5,715 phishing samples, eliminating the need for synthetic minority oversampling and ensuring that accuracy remains a meaningful evaluation metric. Figure 1 presents the class distribution as a radial chart. A comprehensive integrity check confirmed the absence of missing values, duplicate rows, and constant columns. The raw URL field was excluded from modeling because the dataset already encodes its lexical signal through 87 derived features and retaining the string would introduce a quasi-identifier capable of causing trivial memorization. The categorical target was transformed via label encoding such that *y*∈{0, 1}, where *y* = 0 denotes a legitimate URL and *y* = 1 denotes a phishing URL.

**Figure 1:**
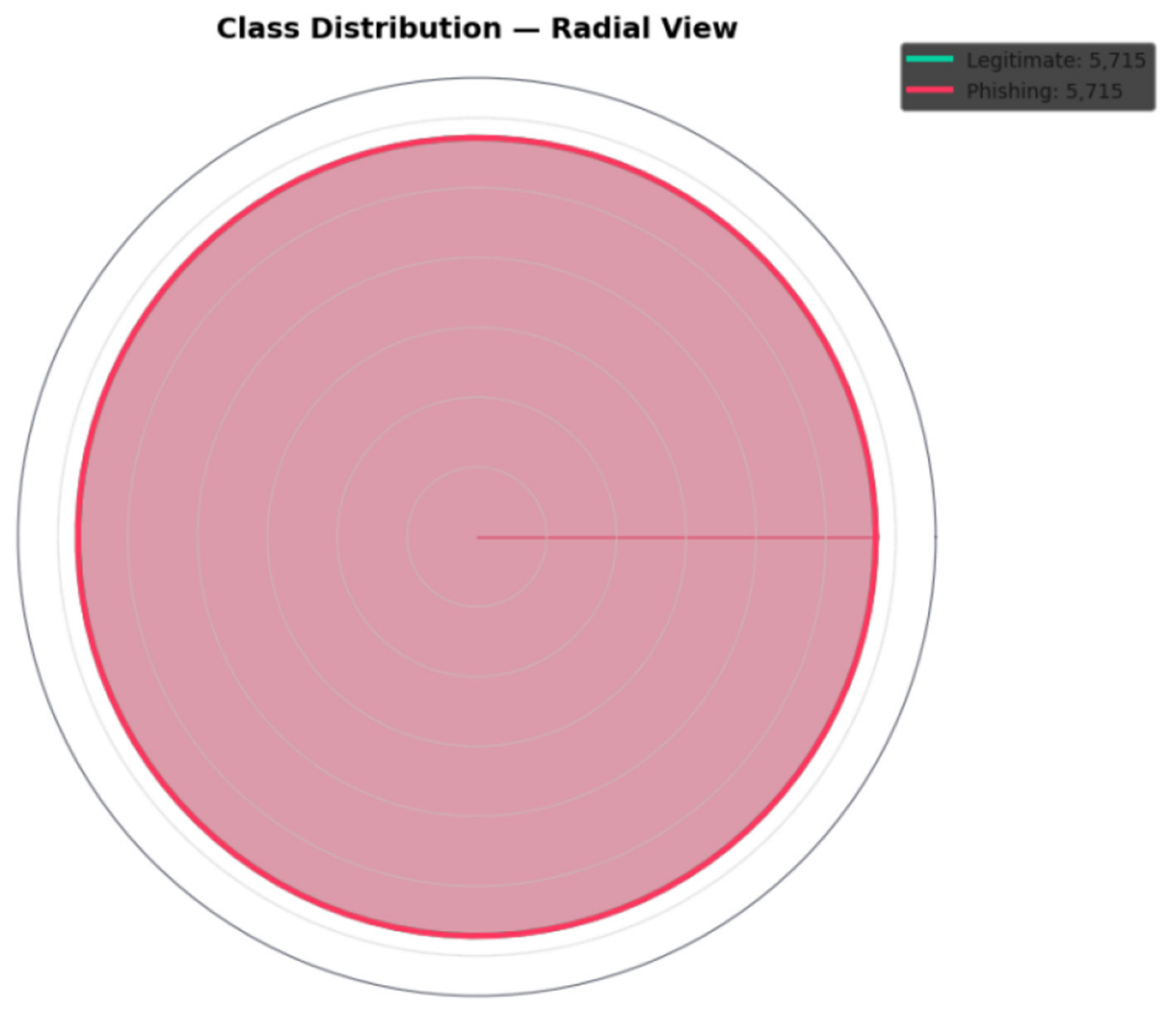
Class distribution—radial view. The dataset is perfectly balanced with 5,715 legitimate (green) and 5,715 phishing (red) URL instances (*N* = 11, 430 total). The near-complete overlap of the two radial traces confirms exact 50:50 balance, eliminating the need for synthetic minority oversampling and ensuring that accuracy remains a valid evaluation metric alongside *F*_1_-score.

### 3.2 Feature taxonomy

To improve interpretability and to organize the downstream explainability analysis, the 87 features were grouped into four conceptual families: (i) URL-lexical descriptors comprising length, character ratios, special-token counts, sub-domain structure, and the presence of shortening services; (ii) HTML-content descriptors comprising hyperlink ratios, anchor safety, iframe presence, and login-form heuristics; (iii) external and WHOIS descriptors comprising domain age, registration length, DNS records, web traffic, page rank, and Google index status; and (iv) a residual “other” group comprising the two features that did not fall into the preceding three families under our keyword taxonomy, namely ip (raw-IP host indicator) and links_in_tags. Neither appears among the top-fifteen ranked attributes under either Tree SHAP or Permutation Importance (Section 4), so the interpretability narrative is unaffected by their heterogeneous nature. This partition is used purely as an analytical lens; it is not encoded as an additional input to any model.

### 3.3 Exploratory data analysis

A multi-faceted exploratory analysis was performed to characterize the marginal and joint structure of the feature space. For every continuous feature *X*_*j*_, the standardized mean gap between the two classes,

$$\begin{array}{l} \Delta_{j}=\frac{\left|\mu_{j}^{\left(0\right)}-\mu_{j}^{\left(1\right)}\right|}{12\left(\sigma_{j}^{\left(0\right)}+\sigma_{j}^{\left(1\right)}\right)+\varepsilon }, \end{array}$$ (1)

was computed to identify the most class-discriminative attributes, where $\mu_{j}^{\left(c\right)}$ and $\sigma_{j}^{\left(c\right)}$ denote the conditional mean and standard deviation of feature *j* in class *c*, and ε = 10^−9^ is a numerical regulariser. Mutual information between each feature and the target,

$$\begin{array}{l} \text{MI}\left(X_{j};Y\right)=\underset{x\in X_{j}}{\sum }\underset{y\in \left\{0,1\right\}}{\sum }p\left(x,y\right)\log\frac{p\left(x,y\right)}{p\left(x\right)p\left(y\right)}, \end{array}$$ (2)

was estimated using the *k*-nearest-neighbor estimator of Kraskov et al. (2004), providing a non-parametric, monotone-invariant ranking of relevance. Higher-order distributional behavior was summarized via Fisher's skewness $\gamma_{1}=E\left[{\left(X-\mu \right)}^{3}\right]/\sigma^{3}$ and excess kurtosis $\gamma_{2}=E\left[{\left(X-\mu \right)}^{4}\right]/\sigma^{4}-3$. Linear redundancy was inspected through Pearson's correlation ρ_*ij*_ = cov(*X*_*i*_, *X*_*j*_)/(σ_*i*_σ_*j*_) restricted to the top-twenty features by mutual information.

Figure 2 presents class-conditional histograms for the 12 most class-discriminative features, Figure 3 shows the top-25 features ranked by mutual information, Figure 4 presents the correlation network of the top-20 MI features, Figure 5 maps the distributional landscape in the skewness-kurtosis plane, and Figure 6 presents the class-conditional radar profile over the top-10 MI features.

**Figure 2:**
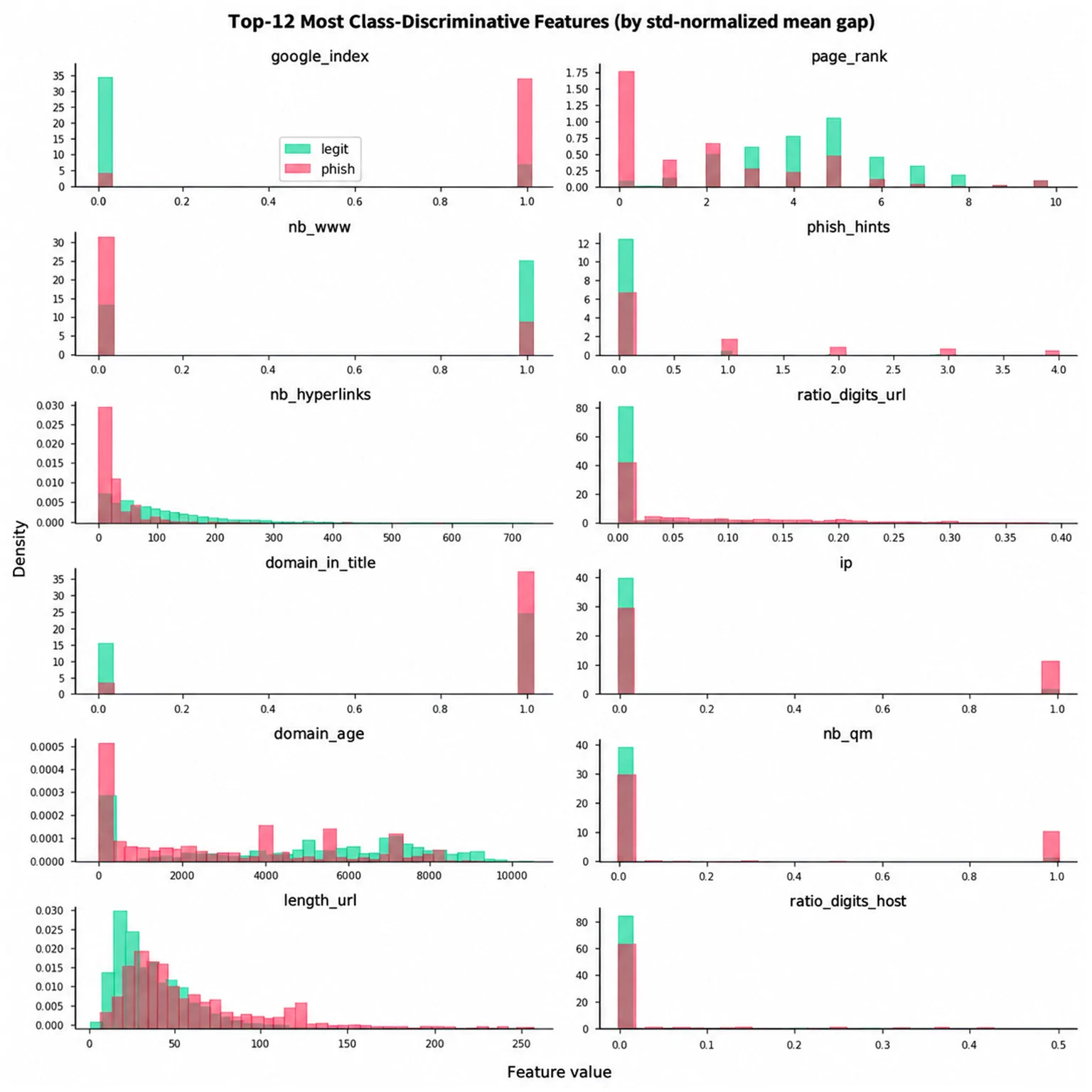
Top-12 most class-discriminative features (by standardized mean gap Δ_*j*_). Class-conditional histograms overlay legitimate (teal) and phishing (pink) distributions for the 12 features with the largest standardized mean-gap score Δ_*j*_ (Equation 1). The external-reputation features google_index and page_rank exhibit the sharpest bimodal separation: Phishing URLs concentrate at low page-rank values and near-total absence from the Google index, while legitimate URLs span a broad positive range. URL-lexical features (length_url, ratio_digits_url) show heavy-tailed phishing distributions. Content features (nb_hyperlinks) exhibit right-skewed legitimate distributions reflecting the richer link structure of authentic pages.

**Figure 3:**
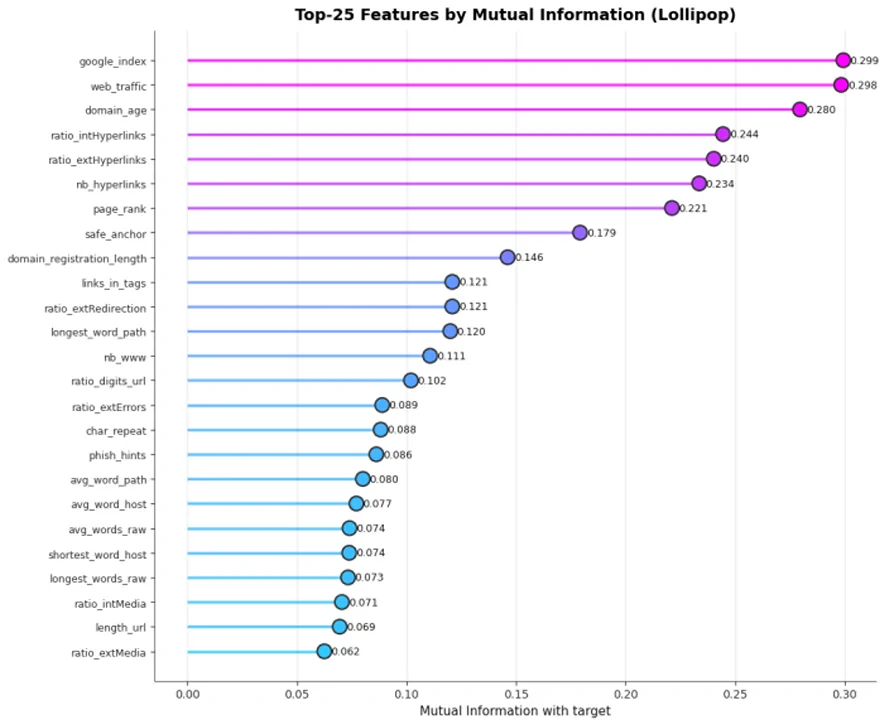
Top-25 features by mutual information with the phishing target (lollipop chart). Features are ranked in descending order of mutual information MI(*X*_*j*_; *Y*) estimated by the *k*-NN estimator (Equation 2). google_index (0.299), web_traffic (0.298), and domain_age (0.280) lead by a substantial margin, confirming that external-reputation signals dominate individual discriminative power. The color gradient (magenta to cyan) encodes MI magnitude. The sharp elbow after rank 8 indicates diminishing informational returns beyond the external-reputation and content-richness feature groups, motivating the mutual-information filter at *k* = 50 applied in the preprocessing pipeline.

**Figure 4:**
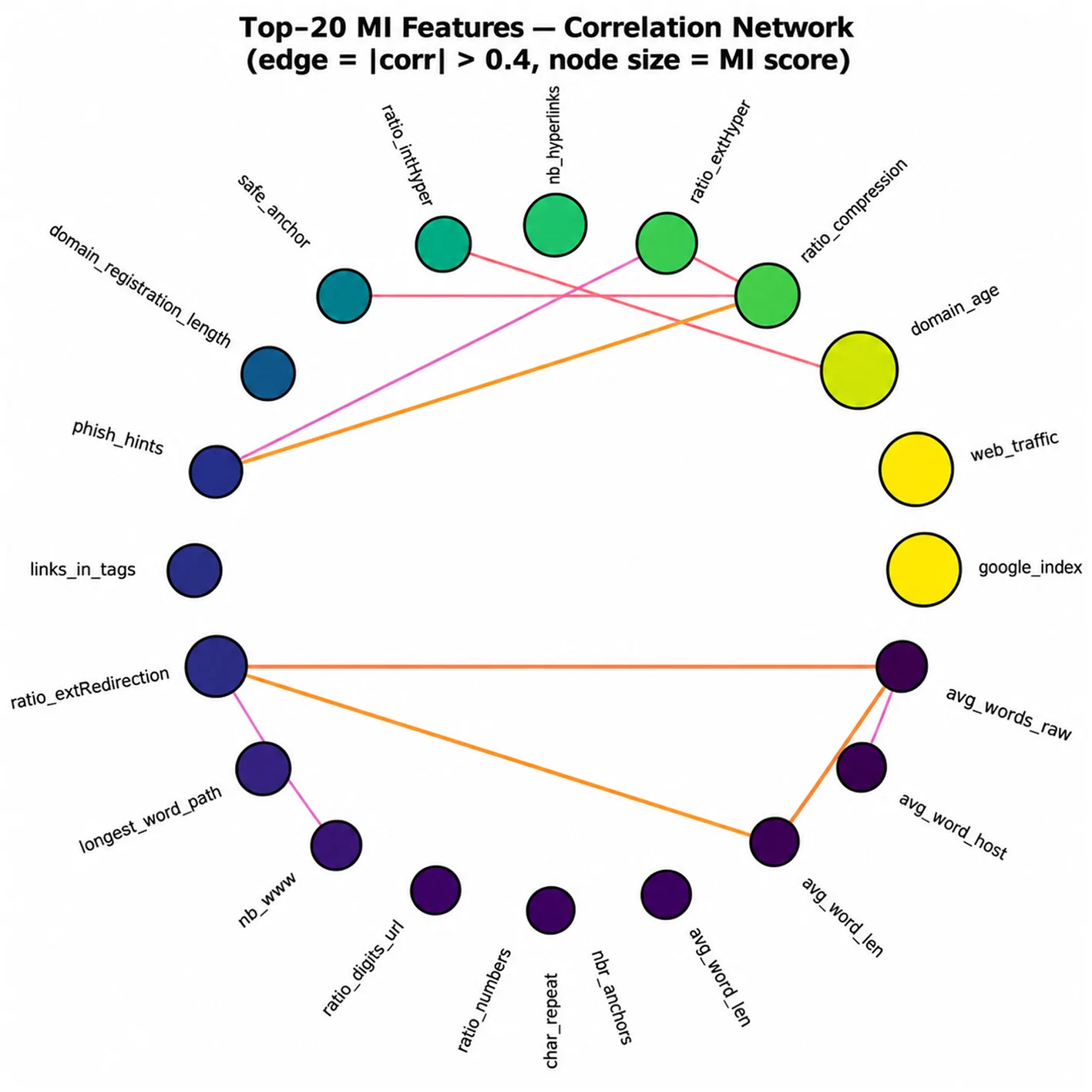
Top-20 MI features—correlation network (edge threshold |ρ|>0.4, node size = MI score). Each node represents one of the 20 highest-MI features; node size encodes MI score, and node color encodes MI rank (yellow = highest). Edges are drawn only for pairs with absolute Pearson's correlation |ρ|>0.4; edge color encodes correlation sign (orange = positive, red = negative). The upper cluster (large yellow/green nodes: web_traffic, domain_age, google_index, nb_hyperlinks, ratio_intHyperlinks, ratio_extHyperlinks) exhibits sparse inter-correlation, indicating that these highly informative features carry largely orthogonal signal and that jointly retaining them adds cumulative value. The lower cluster (nb_www, ratio_digits_url, char_repeat, avg_words_raw, avg_word_host) shows denser redundancy patterns, identifying candidates for removal if a more aggressive feature budget is required.

**Figure 5:**
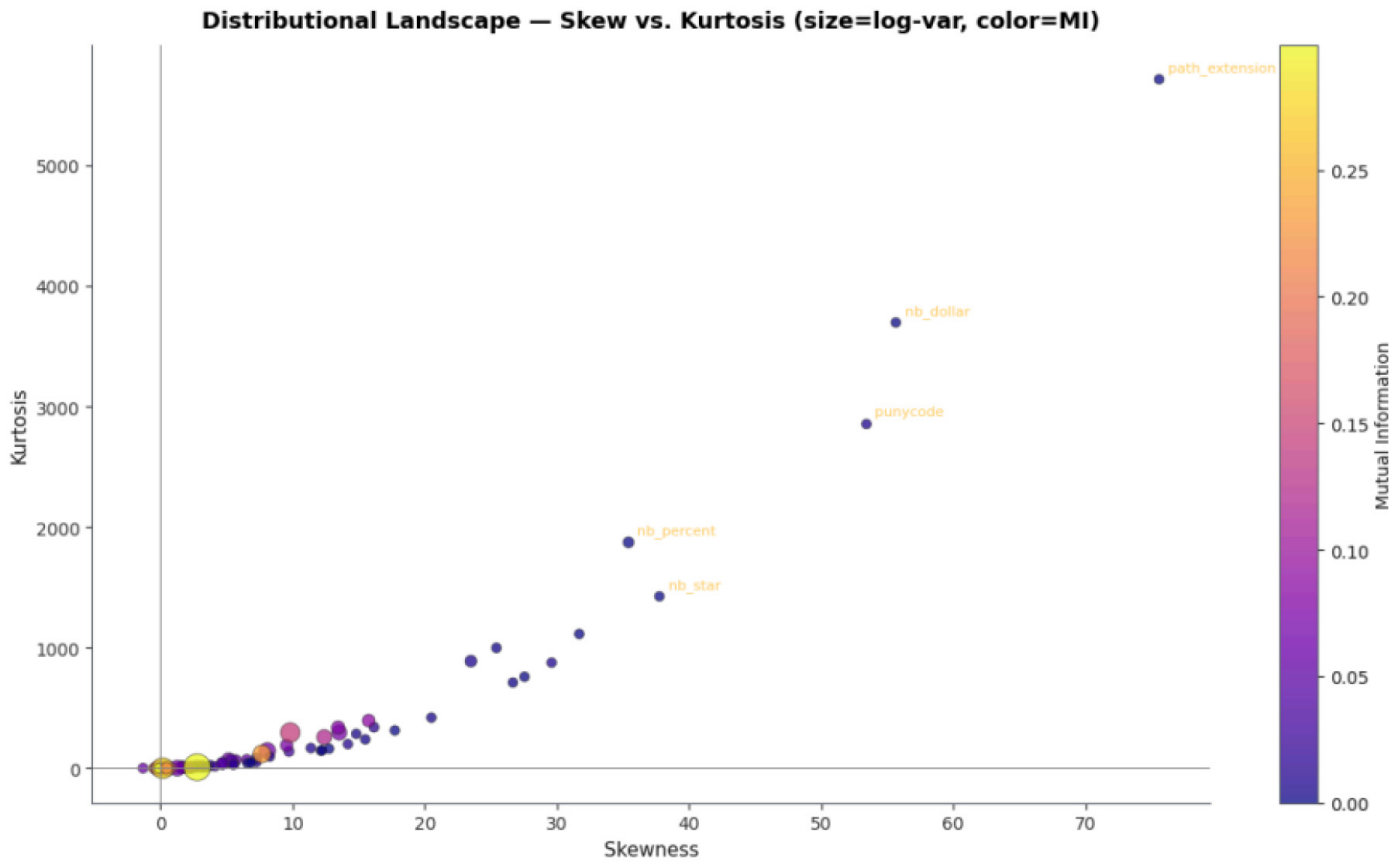
Distributional landscape—skewness vs. kurtosis (size = log-variance, color = MI). Each point represents one of the 87 features plotted by its Fisher skewness (γ_1_, *x*-axis) and excess kurtosis (γ_2_, *y*-axis). Point size encodes log-variance, and point color encodes mutual information (yellow = high MI, purple = low MI). The majority of features cluster in the low-skewness, low-kurtosis region, consistent with approximately symmetric distributions. Extreme outliers (path_extension, nb_dollar, punycode, nb_percent, nb_star) exhibit skewness above 35 and kurtosis above 1,000, reflecting near-binary indicator variables with heavy zero-inflation. These extreme distributional shapes motivate the mutual-information filter over variance-threshold selection since high variance alone (large circle size) does not imply high predictive relevance (low MI, purple color) for several outlier features. This also explains why these high-skewness, high-kurtosis features (path_extension, nb_dollar, punycode, nb_percent, nb_star) never appear among the top-ranked SHAP or Permutation Importance features in Section 4: they are near-binary, heavily zero-inflated indicators that fire on a vanishingly small fraction of URLs, so despite their large variance they carry little mutual information with the target and contribute negligibly to the model's decisions. Their exclusion from the top rankings is thus the expected and desired behavior of a relevance-based selector, not an oversight.

**Figure 6:**
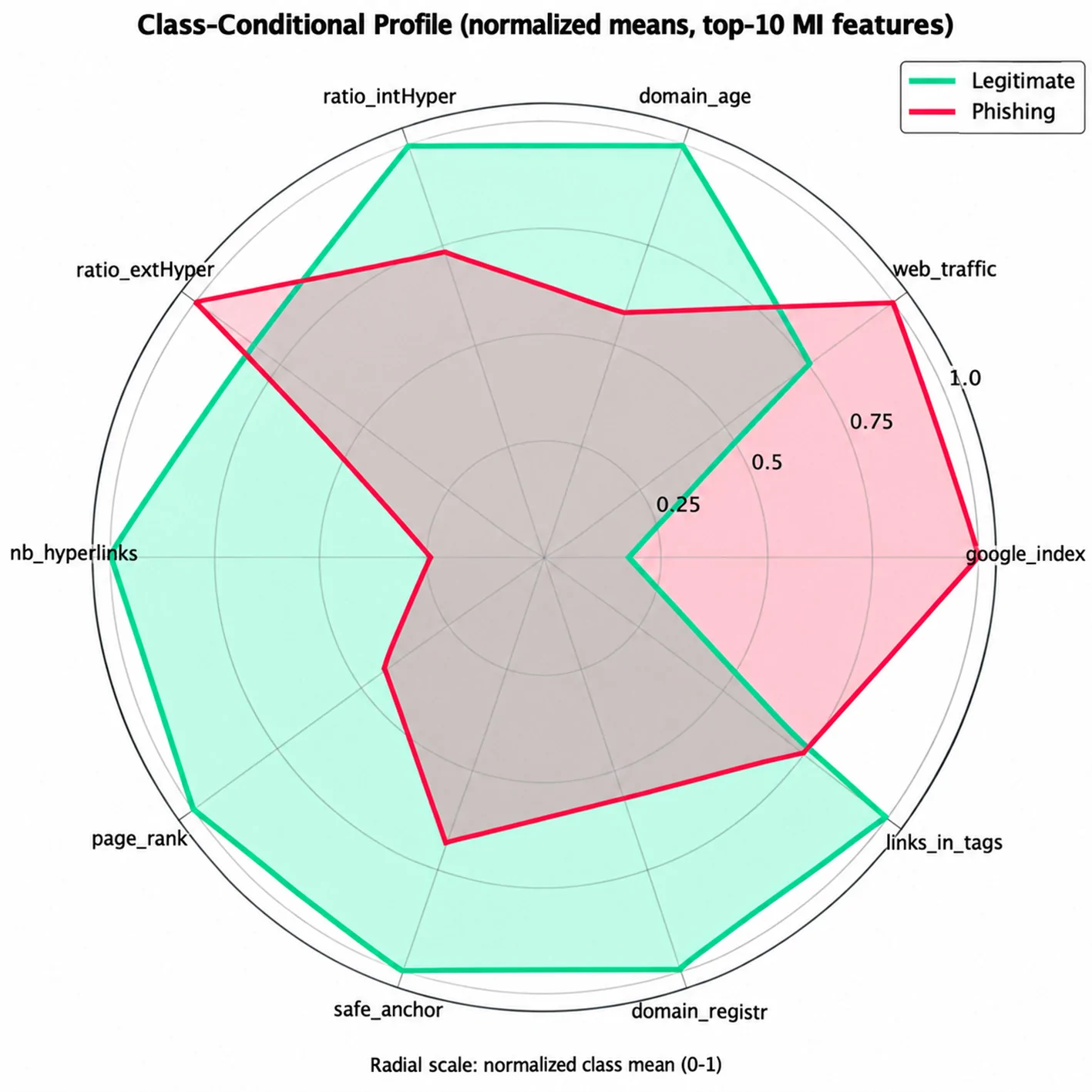
Class-conditional profile—radar chart (normalized means, top-10 MI features). Each axis represents one of the 10 highest-MI features, normalized to [0, 1] by per-feature min-max scaling. The legitimate-URL polygon (teal) is substantially larger than the phishing polygon (red) across the reputation-based axes (domain_age, web_traffic, google_index, page_rank, safe_anchor, nb_hyperlinks, domain_registr), confirming the dominance of external-reputation signals as discriminators of URL authenticity. The phishing polygon exceeds the legitimate one on the ratio_extHyper axis, indicating a higher fraction of external hyperlinks in phishing pages—a content-structure signal complementary to the reputation axes. The visual contrast between the two polygons directly motivates the two-axis narrative (*trust-by-reputation* vs. *risk-by-lexical-complexity*) developed in the discussion.

### 3.4 Train–test partitioning and leakage control

The corpus was split into training and test partitions in an eighty-to-twenty ratio with stratification on the target, yielding *N*_tr_ = 9, 144 and *N*_te_ = 2, 286. To preclude any form of information leakage (Kaufman et al., 2012), all preprocessing transformers were fit exclusively on the training partition and only applied (not refit) to the held-out test set. Feature standardization, given for feature *j* by Equation 3,

$$\begin{array}{l} z_{ij}=\frac{x_{ij}-{\hat{\mu }}_{j}^{\text{(tr)}}}{{\hat{\sigma }}_{j}^{\text{(tr)}}}, \end{array}$$ (3)

used training-set moments only. Univariate filter-based feature selection was then carried out with the top-*k* mutual-information criterion (*k* = 50) computed on the training fold, retaining 50 of the 87 standardized features. The same fitted scaler and selector were subsequently applied to the test fold, guaranteeing that no test-set statistic ever influenced the learned representation.

### 3.5 End-to-end framework overview

Figure 7 presents the complete end-to-end pipeline, illustrating all 11 stages from dataset collection through SHAP and Permutation Importance analysis.

**Figure 7:**
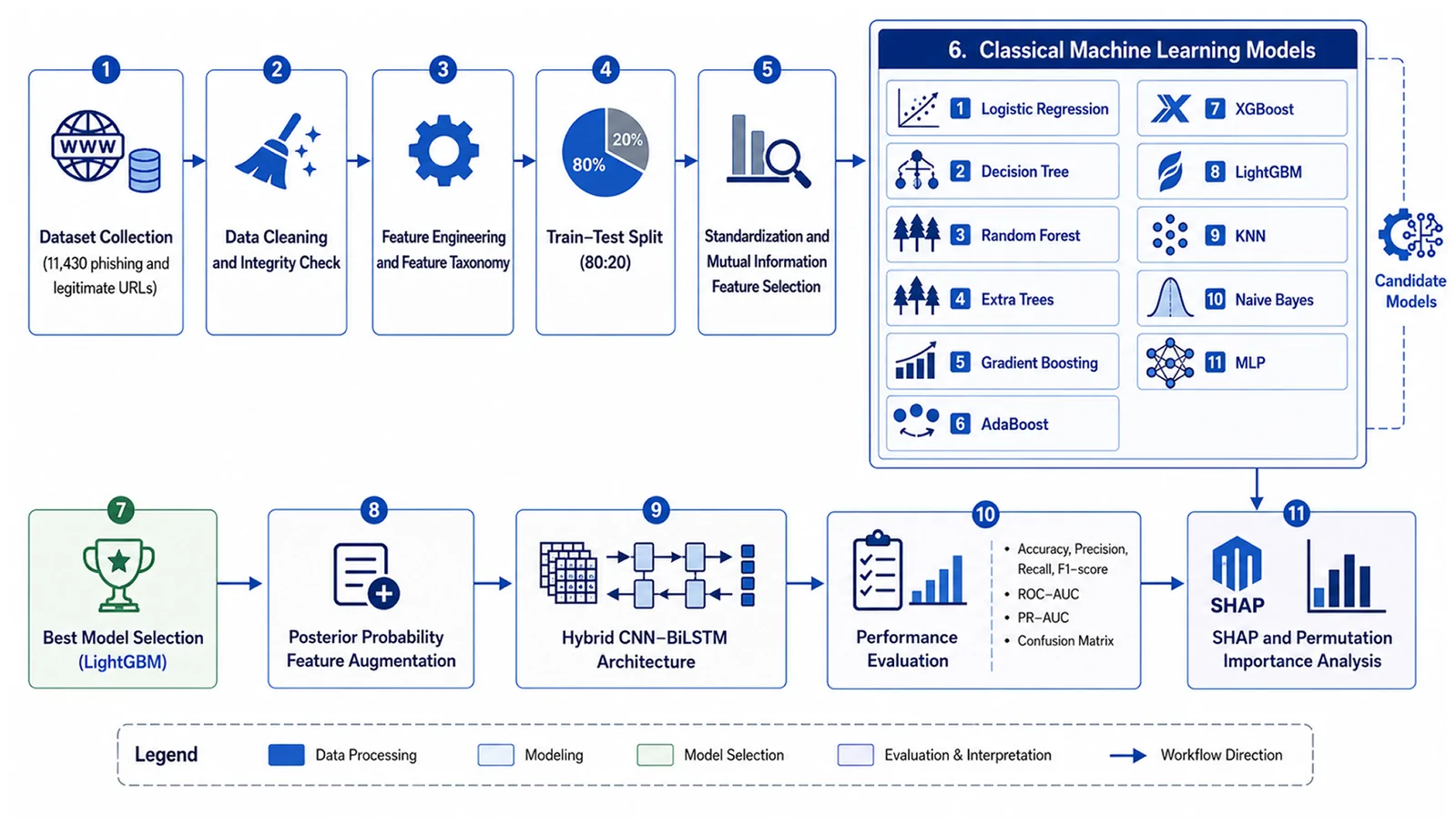
End-to-end machine learning framework for phishing URL detection. The pipeline comprises 11 sequential stages: (1) Dataset collection (11,430 phishing and legitimate URLs); (2) Data cleaning and integrity check; (3) Feature engineering and feature taxonomy into four families; (4) Stratified 80:20 train-test split; (5) Standardization and mutual-information feature selection (top-50 features, fit on training partition only); (6) Eleven candidate classical classifiers (Logistic Regression, Decision Tree, Random Forest, Extra Trees, Gradient Boosting, AdaBoost, XGBoost, LightGBM, KNN, Naive Bayes, and MLP); (7) Best-model selection (LightGBM); (8) Posterior-probability feature augmentation; (9) Hybrid CNN–BiLSTM architecture; (10) Performance evaluation (accuracy, precision, recall, *F*_1_, ROC-AUC, PR-AUC, and confusion matrix); (11) SHAP and Permutation Importance explainability analysis. Color coding: dark blue = data processing; light blue = modeling; green = model selection; gray = evaluation and interpretation.

### 3.6 Classical learning algorithms

Eleven heterogeneous classifiers were trained on the reduced training matrix **X**^(tr)^∈ℝ^9144 × 50^ to provide broad coverage of inductive biases. The pool comprises Logistic Regression, Decision Tree, Random Forest (Breiman, 2001), Extra Trees (Geurts et al., 2006), Gradient Boosting (Friedman, 2001), AdaBoost (Freund and Schapire, 1997), XGBoost (Chen and Guestrin, 2016), LightGBM (Ke et al., 2017), *k*-Nearest Neighbors (Cover and Hart, 1967), Gaussian Naive Bayes, and a Multi-Layer Perceptron. For completeness, we additionally benchmark a Support Vector Machine with a radial-basis-function kernel (*C* = 1, γ = scale). On the selected 50-feature representation, the RBF-SVM is computationally tractable (single fit in 6.7 s) and attains *F*_1_ = 0.9503 and AUC = 0.9879 on the test partition, placing it below all four tree-ensemble methods and confirming the general finding that margin-based kernels do not outperform gradient-boosted trees on heavy-tailed, zero-inflated tabular features (Grinsztajn et al., 2022). We therefore include the RBF-SVM as a reference point but do not carry it into the deep-hybrid or explainability stages, which focus on the strongest classical model. For the gradient-boosted ensembles, the additive model of Equation 4

$$\begin{array}{l} F_{M}\left(x\right)=\overset{M}{\underset{m=1}{\sum }}\nu h_{m}\left(x;\theta_{m}\right),\;\;h_{m}\in H_{\text{tree}}, \end{array}$$ (4)

was optimized under the regularized logistic objective of Equation 5,

$$\begin{array}{l} L\left(\phi \right)=\overset{N_{\text{tr}}}{\underset{i=1}{\sum }}\ell \left(y_{i},{ŷ}_{i}\right)+\overset{M}{\underset{m=1}{\sum }}\Omega \left(h_{m}\right),\;\Omega \left(h\right)=\gamma T+\frac{1}{2}\lambda {\left\|w\right\|}_{2}^{2}, \end{array}$$ (5)

where *T* is the number of leaves, **w** the leaf-weight vector, and ν the shrinkage. The Multi-Layer Perceptron was trained with Adam (Kingma and Ba, 2015) under the binary cross-entropy loss of Equation 6,

$$\begin{array}{l} L_{\text{BCE}}=-\frac{1}{N}\overset{N}{\underset{i=1}{\sum }}\left[y_{i}\log{ŷ}_{i}+\left(1-y_{i}\right)\log\left(1-{ŷ}_{i}\right)\right]. \end{array}$$ (6)

Each model was evaluated by stratified 5-fold cross-validation on the training partition and additionally re-fit on the entire training partition to produce held-out test predictions.

### 3.7 Performance metrics

Letting TP, TN, FP, *and*FN denote the entries of the confusion matrix, the following quantities were reported for every model: accuracy and precision (Equation 7), recall and *F*_1_-score (Equation 8), and the area under the ROC curve (Equation 9):

$$\begin{array}{l} \text{Accuracy}=\frac{\text{TP}+\text{TN}}{\text{TP}+\text{TN}+\text{FP}+\text{FN}},\;\;\text{Precision}=\frac{\text{TP}}{\text{TP}+\text{FP}}, \end{array}$$ (7)

$$\begin{array}{l} \text{Recall}=\frac{\text{TP}}{\text{TP}+\text{FN}},\;\;F_{1}=\frac{2\text{Precision}\cdot \text{Recall}}{\text{Precision}+\text{Recall}}, \end{array}$$ (8)

$$\begin{array}{l} \text{AUC}=\overset{1}{\underset{0}{\int }}\text{TPR}\left({\text{FPR}}^{-1}\left(u\right)\right)du. \end{array}$$ (9)

The *F*_1_-score served as the primary selection criterion because it preserves the equilibrium between false alarms, which erode user trust, and missed detections, which directly cause security breaches.

### 3.8 Statistical significance testing via the Wilcoxon signed-rank test

To verify that the observed performance differences among classifiers are not attributable to fold-level sampling variance, an additional 10-fold stratified cross-validation was executed on the training partition. The slow Gradient Boosting, AdaBoost, and Multi-Layer Perceptron variants were excluded from this stage on grounds of computational expense, having already underperformed in the 5-fold round. Let $a_{i}^{\left(A\right)}$ and $a_{i}^{\left(B\right)}$ denote the *F*_1_-scores of two competing models on fold *i*, *i* = 1, …, 10, and let $d_{i}=a_{i}^{\left(A\right)}-a_{i}^{\left(B\right)}$. The Wilcoxon signed-rank statistic of Equation 10,

$$\begin{array}{l} W=\underset{i:d_{i}\neq 0}{\sum }\text{sgn}\left(d_{i}\right)R_{i},\;\;R_{i}=\text{rank}\left(\left|d_{i}\right|\right), \end{array}$$ (10)

is distribution-free and tests the null hypothesis *H*_0_:median(*d*) = 0 (Wilcoxon, 1945; Demšar, 2006). The reference model was selected as the one with the highest mean fold *F*_1_, and pairwise tests were conducted at α = 0.05. Because the eight retained models give rise to multiple pairwise comparisons against the reference, raw *p*-values were adjusted using the Holm–Bonferroni step-down correction (Holm, 1979) to control the family-wise error rate; both raw and adjusted values are reported. To complement the hypothesis tests with magnitude information, two distribution-free effect sizes were computed for each pairwise comparison: the matched-pairs rank-biserial correlation derived from the signed-rank statistic, and Cliff's delta (Cliff, 1993). Finally, 95% confidence intervals for each model's mean fold *F*_1_ were obtained from the 10-fold scores using the bias-corrected and accelerated bootstrap (Efron, 1987) with 10, 000 resamples. We emphasize that this significance analysis is conducted only over the eight models carried into the 10-fold stage; the Gradient Boosting, AdaBoost, and Multi-Layer Perceptron variants excluded above on computational grounds are therefore outside its scope, and the corresponding claims in this study are framed accordingly.

### 3.9 Feature-extractor construction from the best classical model

The best-performing classical model on the test partition, namely, LightGBM, was repurposed as a deterministic feature extractor for the deep architecture. Given an instance **x**∈ℝ^50^, the augmented representation **Φ**(**x**)∈ℝ^52^ was constructed as

$$\begin{array}{l} \Phi \left(x\right)=\left[x∥{\hat{p}}_{0}\left(x\right)∥{\hat{p}}_{1}\left(x\right)\right], \end{array}$$ (11)

where ∥ denotes concatenation and ${\hat{p}}_{c}\left(x\right)$ is the class-conditional posterior produced by the LightGBM ensemble. Figure 8 illustrates this augmentation pipeline schematically.

**Figure 8:**
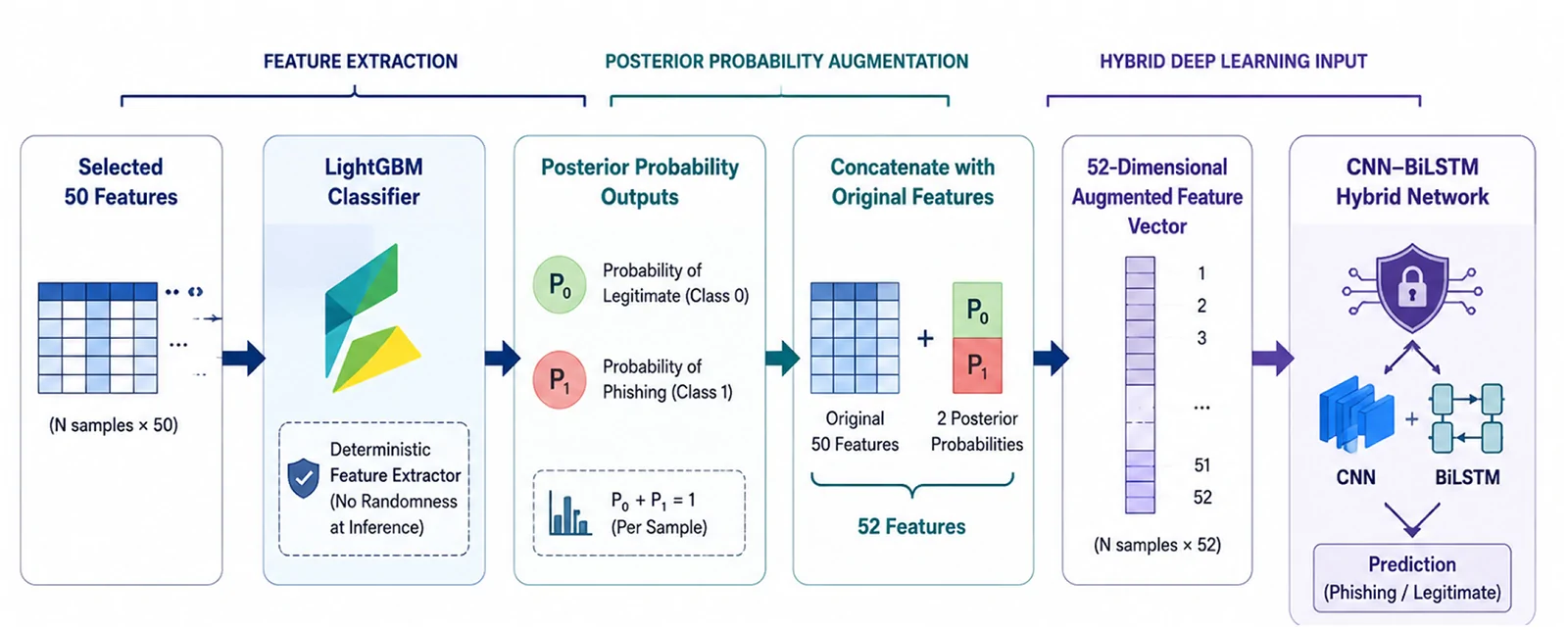
LightGBM posterior probability augmentation pipeline. The 50 mutual-information-selected features (left) are fed to the LightGBM classifier acting as a deterministic feature extractor. The classifier outputs two posterior probabilities: *P*_0_ (probability of legitimate, Class 0) and *P*_1_ (probability of phishing, Class 1), with *P*_0_+*P*_1_ = 1 per sample. These two scalar outputs are concatenated with the original 50-feature vector to produce a 52-dimensional augmented feature vector **Φ**(**x**)∈ℝ^52^ (Equation 11), which constitutes the input to the CNN–BiLSTM hybrid network. The augmentation injects a highly discriminative, non-linear compression of the boosted-tree decision surface into the deep network, providing it with a richer initial representation than the raw features alone.

A subtle but important source of leakage must be controlled at this step. If the posterior probabilities used to augment a training instance were produced by a LightGBM model that had itself been trained on that same instance, the augmented feature would encode the label through the model's near-perfect fit and would constitute stacking leakage, inflating the deep network's apparent performance. To preclude this, the posterior augmentations for the training partition were generated by *out-of-fold* prediction: The training partition was divided into ten folds, and for each fold, the LightGBM posteriors were produced by a model trained only on the other nine folds, so that no instance ever received a posterior from a model that had seen it. The augmentations for the held-out test partition were generated by a LightGBM model fit on the entire training partition and applied to the untouched test set. The mutual-information feature selector and the standardiser were likewise fit only within the appropriate training folds, so the entire augmentation pipeline is free of train–test and stacking leakage. No nested cross-validation of hyperparameters was performed within the out-of-fold loop; LightGBM hyperparameters were fixed at the values selected on the training partition prior to augmentation, which we note as a mild optimism that does not affect the train–test separation. This out-of-fold protocol is the one used to produce the hybrid results reported in Section 4; we verified that replacing in-sample posteriors with out-of-fold posteriors changes the hybrid's test *F*_1_ only marginally (from the in-sample value to 0.9652), confirming that the reported performance does not rest on stacking leakage.

### 3.10 The CNN–BiLSTM hybrid architecture

The augmented vectors were reshaped to **X**∈ℝ^*N*×52 × 1^, treating the feature axis as a one-dimensional sequence. We make explicit that this reshaping imposes a sequential interpretation on what is fundamentally an unordered tabular feature vector: The convolutional kernels act on locally adjacent feature positions and the bidirectional LSTM models order-dependent transitions along the feature axis, yet the ordering of the 50 selected features (mutual-information rank, followed by the two appended posteriors) carries no intrinsic semantic adjacency. The architecture should therefore be understood as using convolution and recurrence as generic parametric mixers over the feature set rather than as exploiting a meaningful sequence, and the empirical gains reported in the ablation study (Section 4.6) are accordingly evidence of useful inductive capacity rather than of a recovered feature order. We did not find that permuting the feature order materially changed performance in preliminary checks, consistent with this interpretation; a systematic study of whether a principled ordering (for example, by hierarchical feature clustering) improves the hybrid is left to future work. The proposed network instantiates two parallel branches that consume this tensor and merge before classification. Figure 9 presents the full architecture diagram.

**Figure 9:**
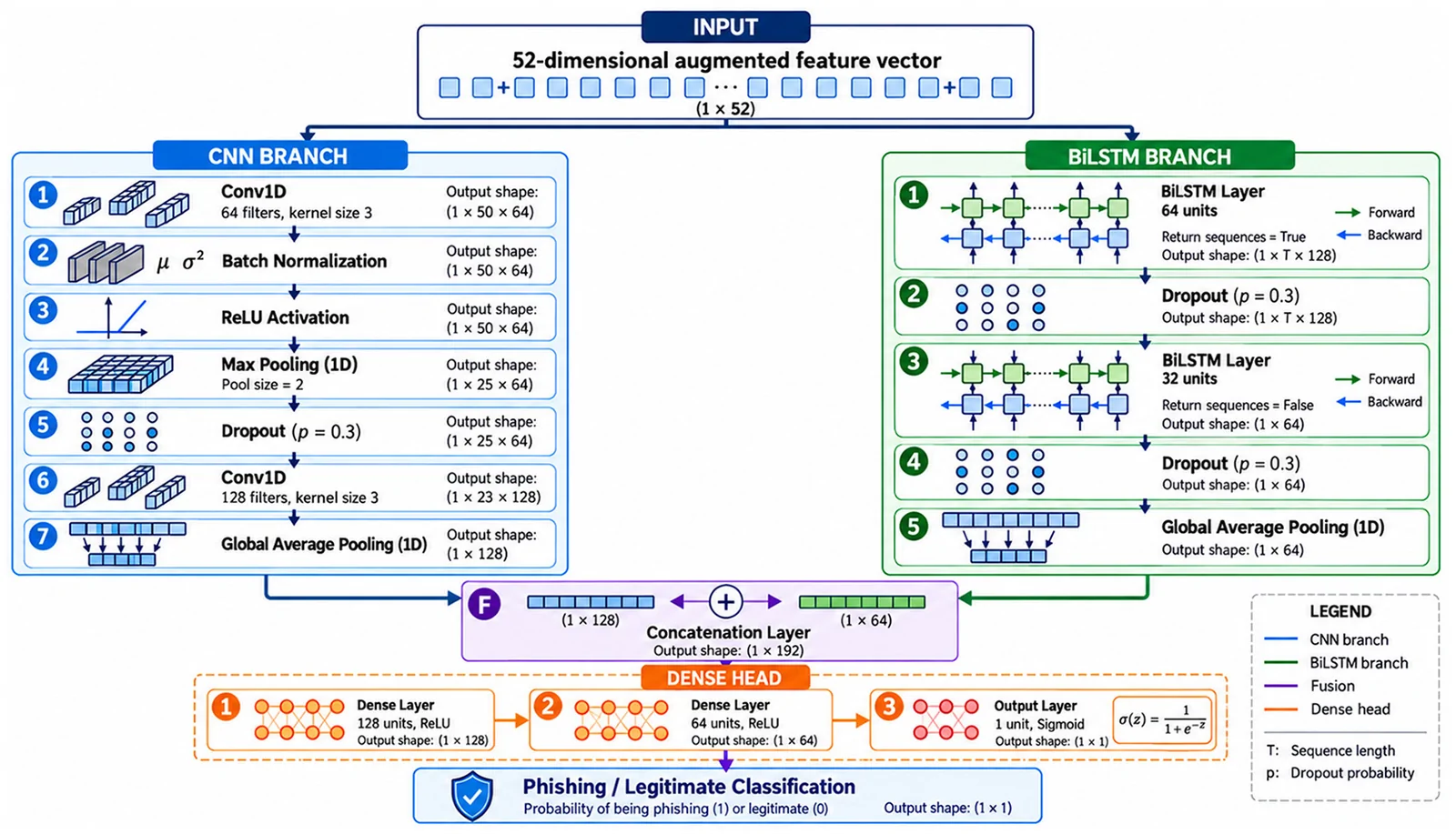
CNN–BiLSTM hybrid architecture for phishing detection. The 52-dimensional augmented feature vector (top) feeds two parallel branches. *CNN Branch* (blue): three convolutional blocks (Conv1D, 64 filters → 64 → 128, kernel size 3), each followed by Batch Normalization, ReLU activation, Max Pooling (stride 2), and Dropout (*p* = 0.3); the branch terminates with Global Average Pooling to a 128-dimensional vector. *BiLSTM Branch* (green): a two-layer bidirectional LSTM (64 units, return sequences; 32 units, final state) with Dropout (*p* = 0.3) between layers and Global Average Pooling to a 64-dimensional vector. *Fusion (F)*: the two branch outputs are concatenated to a 192-dimensional joint embedding and passed through a Dense Head (128 → 64 → 1 units, ReLU activations, Dropout 0.4/0.3, Sigmoid output) that yields the phishing probability. Total trainable parameters: 146,113.

#### 3.10.1 Convolutional branch

A stack of one-dimensional convolutional blocks captures local feature patterns, with the ℓ-th layer computing the feature map of Equation 12:

$$\begin{array}{l} h_{:,c^{\prime }}^{\left(\ell +1\right)}=\text{ReLU}\left(\overset{C_{\ell }}{\underset{c=1}{\sum }}\overset{K-1}{\underset{k=0}{\sum }}W_{k,c,c^{\prime }}^{\left(\ell \right)}h_{:+k,c}^{\left(\ell \right)}+b_{c^{\prime }}^{\left(\ell \right)}\right), \end{array}$$ (12)

with kernel size *K* = 3, *C*_1_ = *C*_2_ = 64, and *C*_3_ = 128 filters, “same” padding, batch normalization (Ioffe and Szegedy, 2015) after each convolution, ReLU activation, max-pooling with stride two, and dropout (Srivastava et al., 2014) of 0.3 between blocks.

#### 3.10.2 Bidirectional LSTM branch

Operating on the same input, a two-layer bidirectional LSTM (Hochreiter and Schmidhuber, 1997; Schuster and Paliwal, 1997) models long-range, order-sensitive interactions across the augmented feature sequence. Each LSTM cell computes the gated recurrence of Equation 13,

$$\begin{array}{l} \begin{array}{cc} i_{t} & =\sigma \left(W_{i}x_{t}+U_{i}h_{t-1}+b_{i}\right),\\ f_{t} & =\sigma \left(W_{f}x_{t}+U_{f}h_{t-1}+b_{f}\right),\\ o_{t} & =\sigma \left(W_{o}x_{t}+U_{o}h_{t-1}+b_{o}\right),\\ {\tilde{c}}_{t} & =\tanh\left(W_{c}x_{t}+U_{c}h_{t-1}+b_{c}\right),\\ c_{t} & =f_{t}⊙c_{t-1}+i_{t}⊙{\tilde{c}}_{t},\\ h_{t} & =o_{t}⊙\tanh\left(c_{t}\right), \end{array} \end{array}$$ (13)

and the bidirectional hidden state is the concatenation ${\vec{h}}_{t}∥{\overset{⃖}{h}}_{t}$. Hidden dimensions are 64 and 32 for the two stacked layers, respectively, with dropout 0.3 between them.

#### 3.10.3 Fusion and classification head

Both branches are reduced by global average pooling to fixed-length vectors, concatenated to a 192-dimensional joint embedding, and passed through a dense block of 128 → 64 → 1 units with ReLU activations and dropouts of 0.4 and 0.3. A sigmoid output produces the phishing probability. The full network has 146,113 trainable parameters.

#### 3.10.4 Optimization

Training minimizes binary cross-entropy with the Adam optimiser at an initial learning rate of 10^−3^, batch size 64, a maximum of 60 epochs, early stopping on validation loss with patience eight, and a learning-rate-on-plateau scheduler with factor 0.5 and patience four. A 15% slice of the training partition served as the validation fold; the test partition was untouched throughout this stage.

### 3.11 Explainable artificial intelligence

Two complementary post-hoc explanation methods were applied to the best classical model so that the explanations are grounded in the most accurate available classifier. Tree SHAP (Lundberg et al., 2020) computes Shapley values ϕ_*j*_ exactly for tree ensembles by attributing the prediction *f*(**x**) to its features as in Equation 14,

$$\begin{array}{lll} f\left(x\right) & = & \phi_{0}+\overset{d}{\underset{j=1}{\sum }}\phi_{j}\left(x\right), \end{array}$$

$$\begin{array}{lll} \phi_{j} & = & \underset{S\subseteq F\backslash \left\{j\right\}}{\sum }\frac{|S|!\left(|F|-|S|-1\right)!}{|F|!}\left[f_{S\cup \left\{j\right\}}\left(x\right)-f_{S}\left(x\right)\right], \end{array}$$ (14)

where *F* is the full feature set and *f*_*S*_ the model conditioned on subset *S*. The mean absolute SHAP value ${\bar{\phi }}_{j}=\frac{1}{n}\underset{i}{\sum }|\phi_{j}\left(x_{i}\right)|$ defines a global ranking, while the signed mean ${\bar{\phi }}_{j}^{\text{sgn}}=\frac{1}{n}\underset{i}{\sum }\phi_{j}\left(x_{i}\right)$ indicates whether a feature pushes predictions toward the phishing class. Permutation Importance is computed as the expected drop in *F*_1_ when feature *j* is independently permuted in the test set, as given in Equation 15:

$$\begin{array}{l} {\text{PI}}_{j}=F_{1}\left(X_{\text{te}},y_{\text{te}}\right)-{𝔼}_{\pi }\left[F_{1}\left(X_{\text{te}}^{\left(\pi_{j}\right)},y_{\text{te}}\right)\right], \end{array}$$ (15)

estimated with 10 random permutations to yield mean and standard deviation. Agreement between SHAP and Permutation rankings within the top-fifteen was measured by intersection cardinality.

### 3.12 Ablation protocol

To isolate the marginal contribution of each major design choice, six ablation variants were defined. (A1) Drops the standardization step and feeds the raw selected features to LightGBM. (A2) Drops the mutual-information feature selection and feeds all 87 features to LightGBM. (A3) Replaces the mutual-information selector with a variance-threshold selector at the same retention budget. (A4) Removes the convolutional branch from the hybrid network, retaining only the BiLSTM branch with the same fusion head. (A5) Removes the BiLSTM branch from the hybrid network, retaining only the convolutional branch. (A6) Removes the LightGBM-derived posterior augmentation and feeds the raw 50-dimensional vectors directly to the dual-branch network. Each variant was trained under the same protocol as the full model, and the resulting test-set *F*_1_-score and AUC are compared in Section 4.6.

### 3.13 Implementation environment

All experiments were implemented in Python 3.12 using scikit-learn 1.4 (Pedregosa et al., 2011), XGBoost 2.0, LightGBM 4.0, TensorFlow 2.15 (Abadi et al., 2016), and SHAP 0.44. Deep-learning training was performed on a single NVIDIA Tesla P100 GPU with 16 GB memory. All random seeds were fixed to 42 for reproducibility. The hyperparameter configurations of the 11 classical models are reported in Table 2 to support full reproducibility; any parameter not listed was left at its library default for the stated version.

**Table 2:** Hyperparameter configurations of the 11 classical classifiers.

Model	Key hyperparameters
Logistic Regression	penalty = ℓ_2_, *C* = 1.0, solver = lbfgs, max_iter = 1000
Decision Tree	criterion = gini, max_depth = None, min_samples_split = 2
Random Forest	n_estimators = 300, max_depth = None, max_features $=\sqrt{d}$, bootstrap = True
Extra Trees	n_estimators = 300, max_depth = None, max_features $=\sqrt{d}$, bootstrap = False
Gradient Boosting	n_estimators = 200, learning_rate = 0.1, max_depth = 3, subsample = 1.0
AdaBoost	n_estimators = 200, learning_rate = 1.0, base = decision stump
XGBoost	n_estimators = 400, learning_rate = 0.1, max_depth = 6, subsample = 0.9, colsample_bytree = 0.9, reg_lambda = 1.0
LightGBM	n_estimators = 400, learning_rate = 0.05, num_leaves = 31, max_depth = −1, subsample = 0.9, colsample_bytree = 0.9, reg_lambda = 1.0
*k*-Nearest Neighbors	n_neighbors = 5, weights = uniform, metric = minkowski (*p* = 2)
Gaussian Naive Bayes	var_smoothing = 10^−9^
Multi-Layer Perceptron	hidden_layers = (128, 64), activation = ReLU, optimiser = Adam, lr = 10^−3^, batch = 64, max_iter = 300

Values for LightGBM, XGBoost, and Extra Trees are the configurations selected by the randomized search; the remaining entries are the fixed defaults used at the baseline stage. Unlisted parameters use library defaults for the versions named in the text. The exact selected values are also serialized in the reproducibility package.

## 4 Results

### 4.1 Exploratory findings

Mutual-information estimates revealed a small set of highly informative features dominating the dependency structure of the corpus. Table 3 reports the 10 most informative features alongside the class-conditional means.

**Table 3:** Ten most informative features, ranked by mutual information with the target.

Rank	Feature	$\mu_{\text{legit}}^{\left(0\right)}$	$\mu_{\text{phish}}^{\left(1\right)}$	MI
1	google_index	0.169	0.899	0.299
2	web_traffic	736,252	977,261	0.298
3	domain_age	5,094	3,031	0.280
4	ratio_intHyperlinks	0.694	0.511	0.244
5	ratio_extHyperlinks	0.250	0.303	0.240
6	nb_hyperlinks	144.32	30.06	0.234
7	page_rank	4.482	1.889	0.221
8	safe_anchor	43.84	30.29	0.179
9	domain_registration_length	624.29	360.77	0.146
10	links_in_tags	59.63	44.32	0.121

Two macroscopic patterns emerge. First, external-reputation signals including Google indexing, Alexa-like web traffic, page rank, domain age, and registration length are by far the most discriminative attributes. Phishing URLs are systematically newer, less-indexed, lower-trafficked, and registered for shorter durations. The Google-index feature alone exhibits a more than 5-fold mean ratio between classes, confirming that search-engine visibility is one of the strongest single indicators of legitimacy. Second, content-richness features such as hyperlink counts, internal/external link ratios, and anchor safety provide an orthogonal signal: Legitimate pages contain on average 144 hyperlinks against only 30 for phishing pages, and a substantially higher proportion of those links point internally. This dual signal of external trust and content density defines the empirical phishing manifold uncovered by the feature space.

### 4.2 Baseline comparison of classical models

Eleven classifiers were trained under identical preprocessing and evaluated on the held-out test partition. Their full performance profiles are summarized in Table 4, ordered by descending *F*_1_. Figure 10 presents the multi-metric leaderboard as a horizontal grouped bar chart.

**Figure 10:**
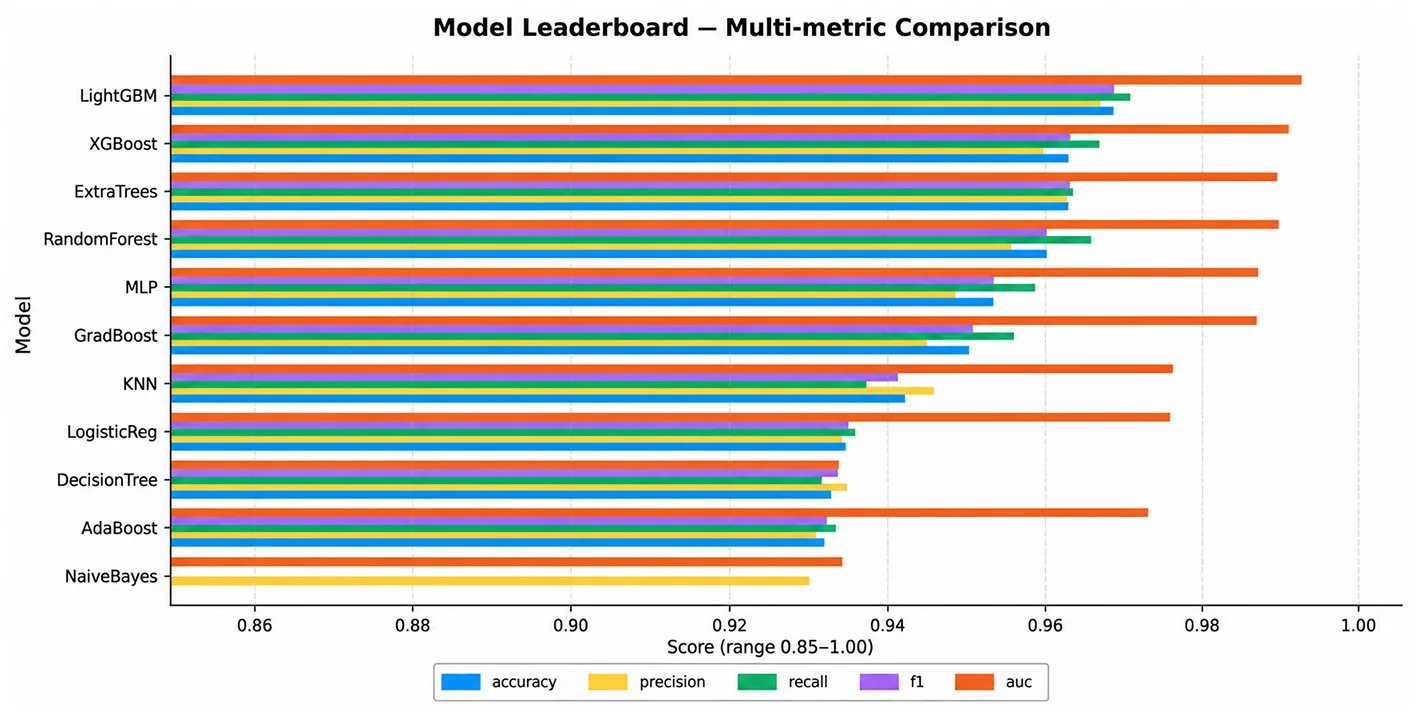
Model leaderboard—multi-metric comparison (score range 0.85–1.00). Horizontal grouped bar chart showing accuracy (blue), precision (yellow), recall (teal), *F*_1_ (purple), and AUC (orange) for all 11 classifiers, ordered by descending AUC. The four tree-ensemble methods (LightGBM, XGBoost, Extra Trees, and Random Forest) cluster at the top of the leaderboard, with all five metrics above 0.96. NaiveBayes is the sole systematic outlier, with recall dropping to 0.56 due to the conditional-independence assumption being violated by the correlated feature space. The zoomed x-axis (0.85–1.00) expands the region of practical interest, making inter-model differences of 1–3 percentage points visually apparent.

**Table 4:** Test-set performance of the eleven classical models.

Model	CV *F*_1_	Acc.	Prec.	Rec.	*F*_1_	AUC	Time (s)
LightGBM	0.9683 ± 0.0055	0.9698	0.9678	0.9720	0.9699	0.9943	1.8
XGBoost	0.9682 ± 0.0080	0.9637	0.9601	0.9676	0.9638	0.9936	3.3
ExtraTrees	0.9617 ± 0.0057	0.9637	0.9633	0.9641	0.9637	0.9928	6.8
RandomForest	0.9634 ± 0.0070	0.9606	0.9558	0.9659	0.9608	0.9933	11.6
MLP	0.9580 ± 0.0058	0.9532	0.9481	0.9589	0.9535	0.9896	18.5
GradBoost	0.9557 ± 0.0087	0.9510	0.9463	0.9563	0.9513	0.9896	13.1
KNN	0.9420 ± 0.0066	0.9418	0.9461	0.9370	0.9415	0.9773	2.3
LogisticReg.	0.9427 ± 0.0076	0.9344	0.9336	0.9353	0.9344	0.9809	3.5
DecisionTree	0.9299 ± 0.0036	0.9335	0.9350	0.9318	0.9334	0.9335	0.7
AdaBoost	0.9461 ± 0.0076	0.9331	0.9319	0.9344	0.9332	0.9849	11.3
NaiveBayes	0.7642 ± 0.0256	0.7612	0.9320	0.5634	0.7023	0.9352	0.2

CV columns report mean and standard deviation across five stratified folds on the training partition.

LightGBM achieves the strongest performance across every metric simultaneously, attaining *F*_1_ = 0.9699 and AUC = 0.9943 on the test partition. The four tree-ensemble methods (LightGBM, XGBoost, Extra Trees, and Random Forest) form a tight performance cluster between *F*_1_ = 0.961 and 0.970 and clearly outperform the linear, kernel-free, instance-based, and shallow-tree alternatives.

The training time reported for LightGBM in Table 4 (1.8 s) is the single-fit cost of the selected configuration at native multithreading, consistent with LightGBM's histogram-based efficiency and comparable to XGBoost (3.3 s). We flag this explicitly because an earlier version of our pipeline logged the full single-threaded grid-search wall-clock time (548.6 s) in this column, which would misleadingly suggest LightGBM is two orders of magnitude slower than XGBoost; that larger figure is the end-to-end model-selection time, not the per-model training cost, and is reported separately. This distinction is material to the deployment discussion in Section 5, where inference-time efficiency, not search time, governs the comparison.

### 4.3 Statistical significance of the performance gap

To verify that the LightGBM advantage is statistically robust rather than the product of fold-level noise, the Wilcoxon signed-rank test was applied to 10-fold *F*_1_-scores collected from eight competing classifiers. The mean fold *F*_1_ values were 0.9698 (LightGBM), 0.9685 (XGBoost), 0.9642 (Random Forest), 0.9626 (Extra Trees), 0.9428 (Logistic Regression), 0.9426 (*k*-Nearest Neighbors), 0.9348 (Decision Tree), and 0.7015 (Naive Bayes). Figure 11 presents the 10-fold CV *F*_1_ distributions as violin plots with Wilcoxon significance annotations. Pairwise comparisons against LightGBM are reported in Table 5.

**Figure 11:**
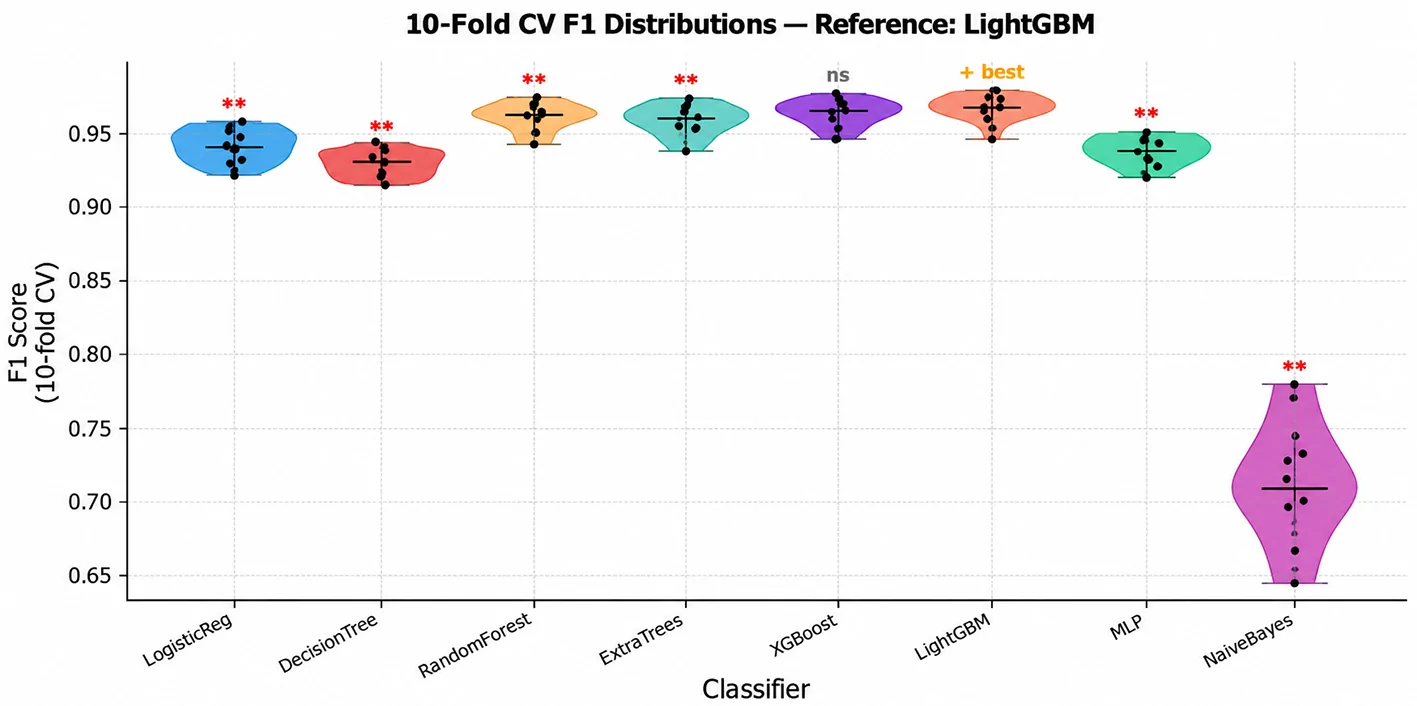
10-fold cross-validation *F*_1_ distributions (violin plots)—reference: LightGBM. Each violin shows the distribution of per-fold *F*_1_ scores (*n* = 10) for one classifier; individual fold observations are overlaid as points. Stars above each violin indicate Wilcoxon signed-rank test significance against LightGBM as reference (^**^p ≤ 0.002, ns *p*>0.05; labeled in red and gold respectively). LightGBM (labeled “best”) achieves the highest median and lowest variance. XGBoost is the only non-significant competitor (“ns”), confirming statistical equivalence (*p* = 0.375). NaiveBayes (rightmost, magenta) is dramatically separated from the remaining classifiers, with median *F*_1_≈0.70 and high fold-to-fold variability. ^**^ *p* ≤ 0.002 (statistically significant after Holm-Bonferroni correction); ns = not significant (*p*> 0.05).

**Table 5:** Wilcoxon signed-rank test of *F*_1_ across 10 cross-validation folds, with LightGBM as the reference model.

Comparison	*W*	*p*_raw_	*p*_Holm_	δ	*r*_rb_	95*%* CI on gap
LightGBM vs. LogisticRegression	0.0	0.0020	0.0137	0.94	+1.000	[0.0242, 0.0299]
LightGBM vs. DecisionTree	0.0	0.0020	0.0137	1.00	+1.000	[0.0306, 0.0399]
LightGBM vs. RandomForest	0.0	0.0020	0.0137	0.47	+1.000	[0.0042, 0.0069]
LightGBM vs. ExtraTrees	0.0	0.0020	0.0137	0.54	+1.000	[0.0049, 0.0094]
LightGBM vs. XGBoost	18.0	0.3750	0.3750	0.11	+0.345	[−0.0004, 0.0031]
LightGBM vs. KNN	0.0	0.0020	0.0137	0.96	+1.000	[0.0239, 0.0305]
LightGBM vs. NaiveBayes	0.0	0.0020	0.0137	1.00	+1.000	[0.2466, 0.2911]

*p*_raw_ is the unadjusted two-sided *p*-value; *p*_Holm_ is the Holm–Bonferroni-adjusted value controlling the family-wise error rate across the seven comparisons. δ is Cliff's delta and *r*_rb_ the matched-pairs rank-biserial correlation (effect sizes). The final column is the 95% bootstrap confidence interval (10, 000 resamples) on the reference–competitor mean-*F*_1_ gap.

After Holm–Bonferroni adjustment, the six significant comparisons remain significant at α = 0.05 (adjusted *p* = 0.0137), and the single non-rejection involving XGBoost is unchanged (*p* = 0.375); the family-wise correction, therefore, does not alter any qualitative conclusion. The effect sizes corroborate this: Cliff's δ and the rank-biserial correlation are large for the six dominated competitors (LightGBM achieved the higher fold *F*_1_ on every one of the ten folds) and small for XGBoost (δ = 0.11, *r*_rb_ = 0.345). Decisively, the 95% bootstrap confidence interval on the LightGBM–XGBoost mean-*F*_1_ gap, [−0.0004, 0.0031] straddles zero, providing direct interval evidence that the two boosting frameworks are statistically equivalent on this corpus, whereas every other interval lies strictly above zero.

LightGBM is statistically superior to six of the seven competitors carried into this stage at α = 0.05. The single non-rejection (*p* = 0.375) involves XGBoost, which echoes the closeness of their point estimates and confirms that the two boosting frameworks are practically interchangeable on this corpus, while both significantly outperform every other family evaluated here. We reiterate that Gradient Boosting, AdaBoost, and the Multi-Layer Perceptron were not carried into the 10-fold stage and are therefore not covered by this significance claim; the broader assertion is accordingly limited to the models actually tested.

### 4.4 The CNN–BiLSTM hybrid

Equipped with the LightGBM-augmented representation **Φ**(**x**)∈ℝ^52^, the proposed CNN–BiLSTM hybrid was trained for at most 60 epochs with early stopping on the validation loss and learning-rate reduction on plateau; the best validation loss was reached after roughly two dozen epochs, and the best weights were restored. Figure 12 presents the training dynamics. The final test-set performance is summarized in Table 6 alongside the unaided LightGBM, and Figure 13 shows the confusion matrix on the test partition. Figure 14 presents the final radar comparison of LightGBM, CNN–BiLSTM, Logistic Regression, and Naive Bayes.

**Figure 12:**
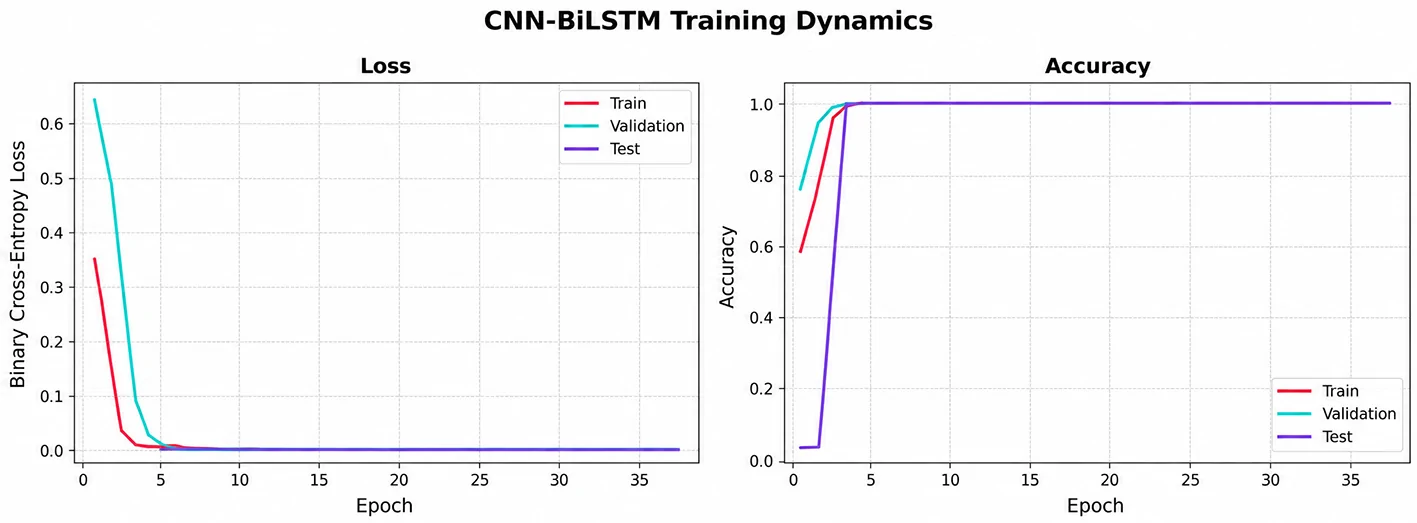
CNN–BiLSTM training dynamics. **Left**: Binary cross-entropy loss on training (red) and validation (cyan) sets across 37 epochs (early stopping triggered at epoch 37). Both losses converge rapidly within the first five epochs and stabilize below 10^−2^; the training–validation gap is negligible, confirming an absence of overfitting. **Right**: Accuracy on training (green) and validation (purple) sets. Full validation accuracy is achieved by epoch four, reflecting the near-linear separability of the LightGBM-augmented feature space and the effectiveness of the posterior-probability augmentation in compressing the discrimination task.

**Table 6:** Comparison of the best classical model and the proposed CNN–BiLSTM hybrid on the held-out test partition.

Model	Accuracy	Precision	Recall	*F*_1_	AUC
LightGBM (best classical)	0.9698	0.9678	0.9720	0.9699	0.9943
CNN–BiLSTM (hybrid)	0.9650	0.9602	0.9703	0.9652	0.9903

**Figure 13:**
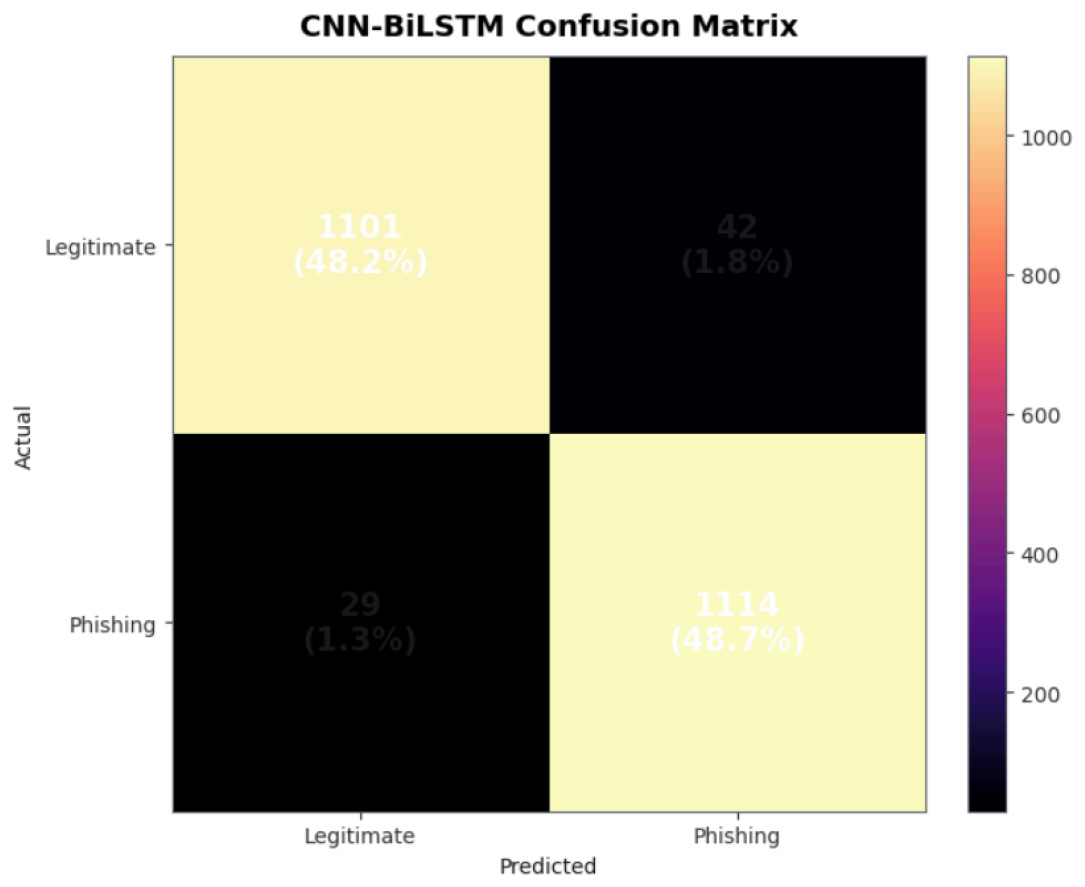
CNN–BiLSTM confusion matrix on the held-out test partition (*n* = 2, 286). Counts on the leakage-safe out-of-fold-augmented hybrid. True Negatives (Legitimate predicted as Legitimate): 1,097 (48.0%); True Positives (Phishing predicted as Phishing): 1,109 (48.5%). False Positives (Legitimate predicted as Phishing): 46 (2.0%); False Negatives (Phishing predicted as Legitimate): 34 (1.5%). The asymmetry in error types (FP > FN) reflects the recall-favorable operating point of the hybrid architecture: The model accepts slightly more false alarms in exchange for a higher true-positive rate, a trade-off of value in security-sensitive deployments where missing a phishing URL incurs a higher cost than an unnecessary warning. Expressed as row-conditional (per-true-class) rates, which are the quantities most relevant to deployment reasoning, the false-positive rate is 46/(1, 097+46) = 4.0% of legitimate URLs and the false-negative rate is 34/(1, 109+34) = 3.0% of phishing URLs, giving a per-class recall of 96.0% for the legitimate class and 97.0% for the phishing class.

**Figure 14:**
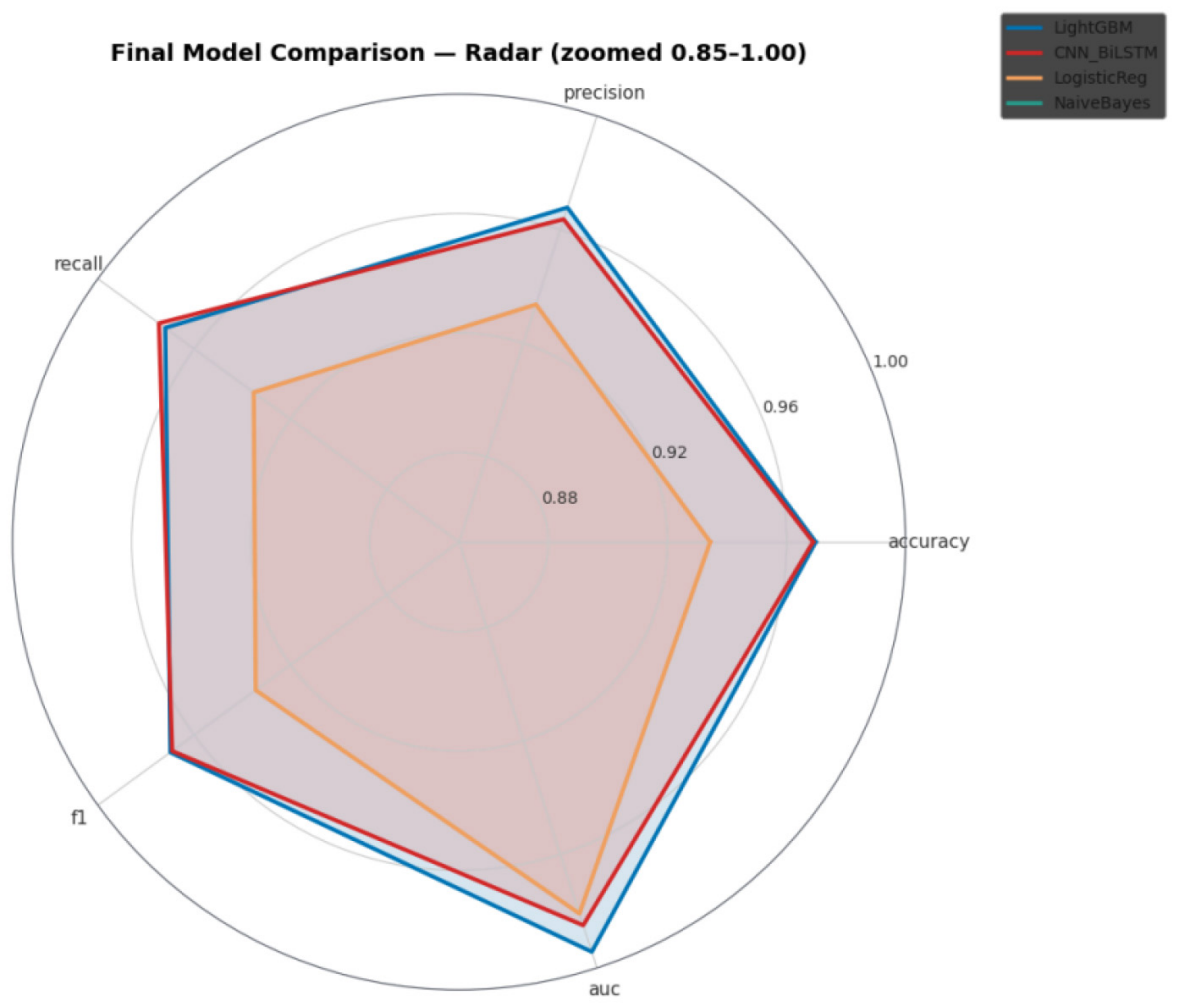
Final model comparison—radar chart (zoomed 0.85–1.00). Each axis represents one evaluation metric (precision, accuracy, *F*_1_, AUC, and recall), with the scale zoomed to [0.85, 1.00] to amplify inter-model differences. LightGBM (blue) and CNN–BiLSTM (red) produce nearly overlapping polygons across all five axes, with CNN–BiLSTM exhibiting a slight recall advantage and LightGBM a slight AUC advantage. Logistic Regression (orange) occupies a smaller polygon interior, underperforming primarily on Precision and *F*_1_. Naive Bayes (teal) exhibits a strongly asymmetric profile driven by its very low recall (0.56), confirming its unsuitability as a standalone phishing detector despite competitive AUC.

The CNN–BiLSTM does not surpass the unaided LightGBM on aggregate *F*_1_ (0.9652 vs. 0.9699), confirming that the deep hybrid is not introduced as a more accurate classifier. Its value lies instead in the precision–recall trade-off: under the leakage-safe out-of-fold augmentation, the hybrid reaches a recall of 0.9703 at a precision of 0.9602, an operating point that favors detection sensitivity. Operationally, this trade is relevant in cybersecurity deployments where the cost of a missed phishing URL exceeds that of an additional false alarm; the hybrid therefore offers a deployable alternative tuned for recall rather than an accuracy improvement over the boosted-tree baseline.

### 4.5 Explainable AI findings

Tree SHAP and Permutation Importance were applied to LightGBM to provide model-faithful explanations. Table 7 reports the top features under each criterion alongside their signed mean SHAP contributions and the implied directional effect. Figure 15 presents the global SHAP importance bar chart, Figure 16 presents the SHAP value distributions per feature, Figure 17 presents the Permutation Importance rankings, and Figure 18 presents the cross-method agreement scatter plot.

**Figure 15:**
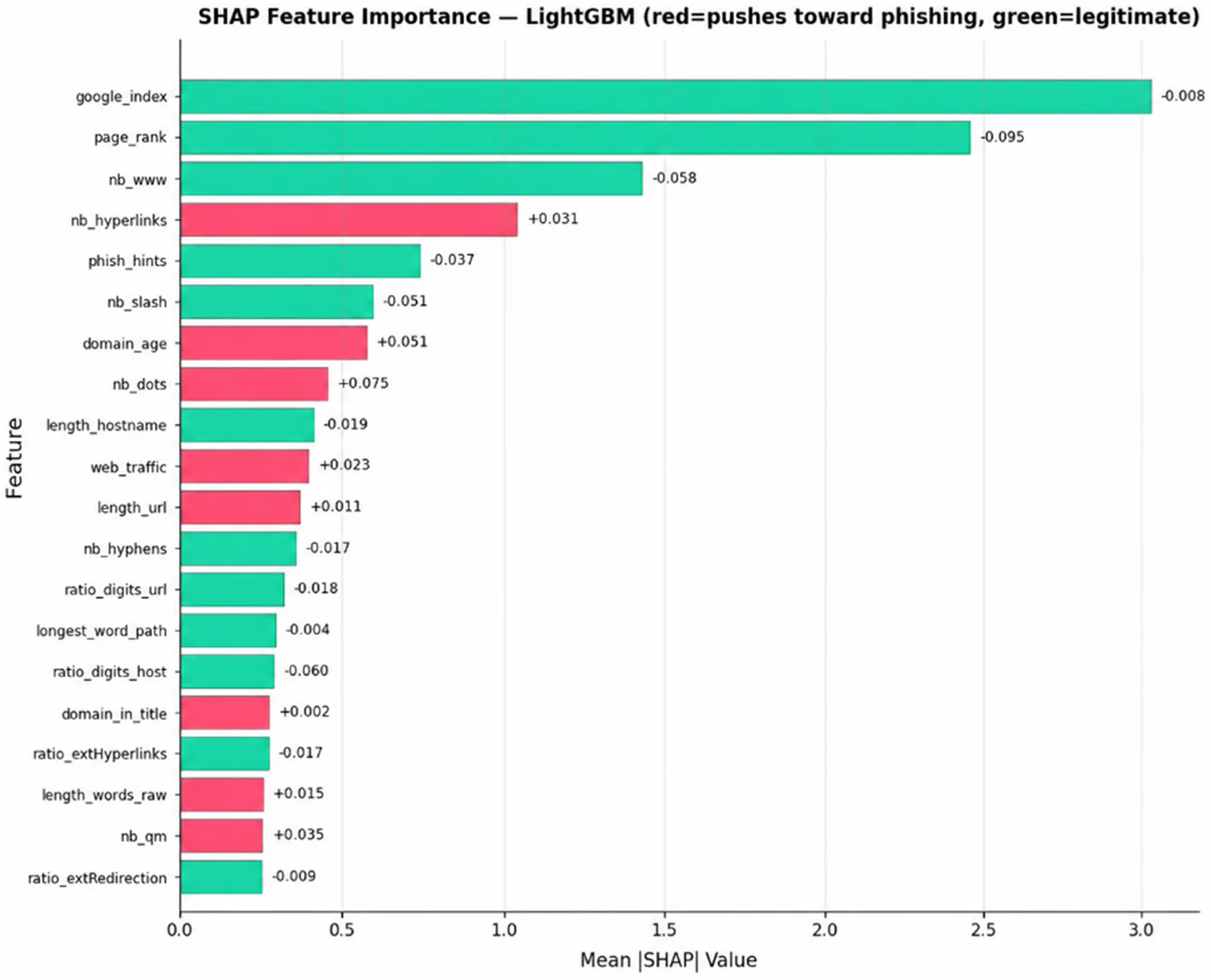
SHAP feature importance—LightGBM (signed mean |SHAP|, red = pushes toward phishing, green = toward legitimate). Features are ranked by mean absolute SHAP value ${\bar{\phi }}_{j}$. google_index and page_rank dominate with ${\bar{\phi }}_{j}$ of approximately 3.0 and 2.5, respectively. The color of each bar encodes the signed mean SHAP (${\bar{\phi }}_{j}^{\text{sgn}}$): Green bars indicate features whose elevation on average pushes predictions toward the legitimate class (negative signed mean, e.g., high page_rank → legitimate), while red bars indicate features pushing toward phishing (positive signed mean, e.g., high nb_dots → phishing). The directional asymmetry cleanly separates the external-reputation group (green: google_index, page_rank, nb_www, nb_hyperlinks, domain_age) from the URL-lexical complexity group (red: phish_hints, nb_dots, nb_hyphens, length_url).

**Figure 16:**
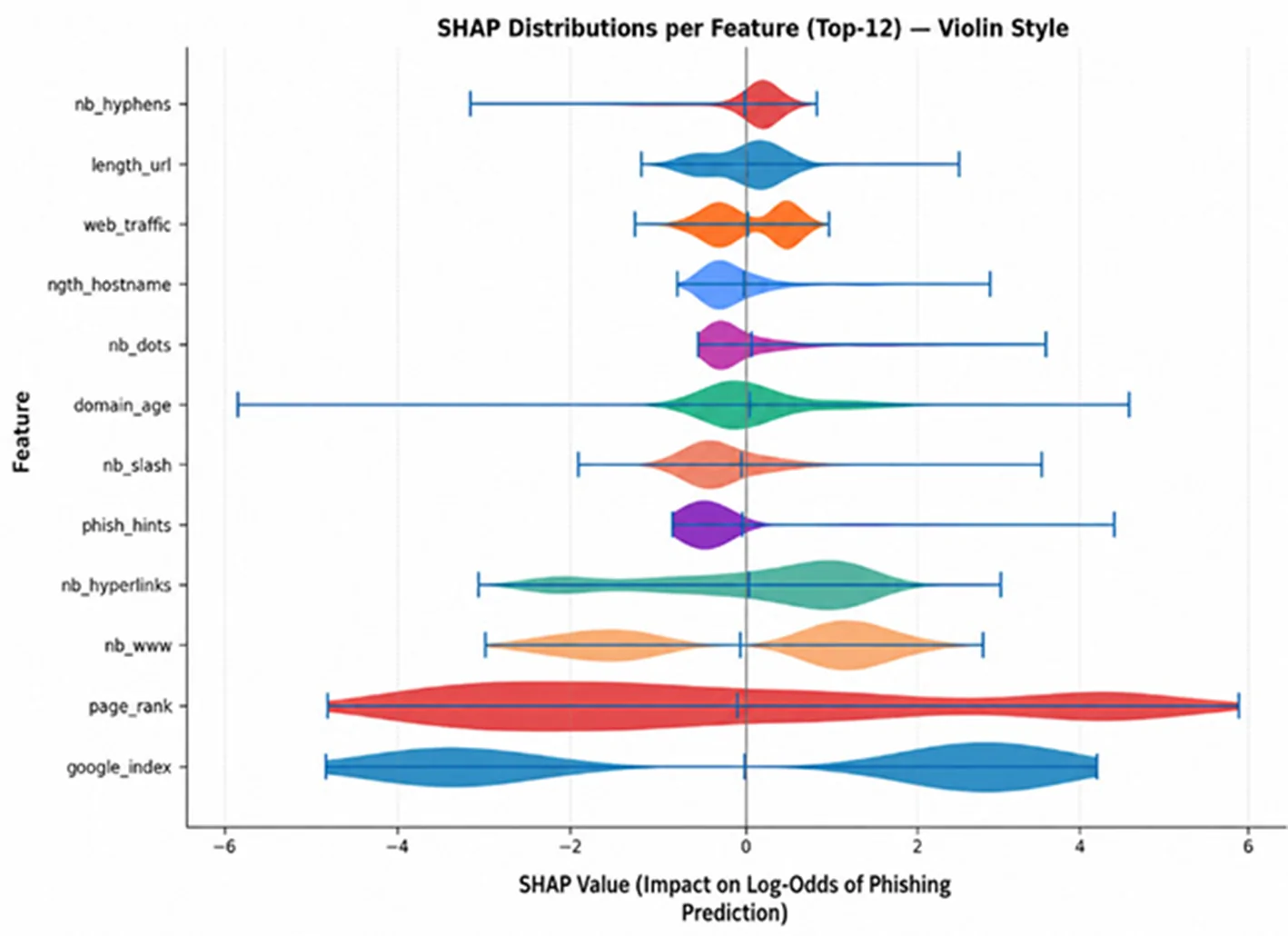
SHAP value distributions per feature (Top-12)—violin style. Each row represents one of the 12 highest-SHAP features; the *x*-axis shows the raw SHAP value (impact on log-odds of phishing prediction). Violin widths encode the kernel density of SHAP values across the test set; horizontal error bars extend to the 5^th^–95^th^ percentile. google_index and page_rank exhibit bimodal SHAP distributions, reflecting their near-binary nature: High google_index contributes a large negative SHAP (toward legitimate), while zero google_index contributes a large positive SHAP (toward phishing). domain_age and phish_hints show heavy-tailed distributions extending beyond ±4, indicating that their extreme values exert very large individual-level influence on predictions.

**Figure 17:**
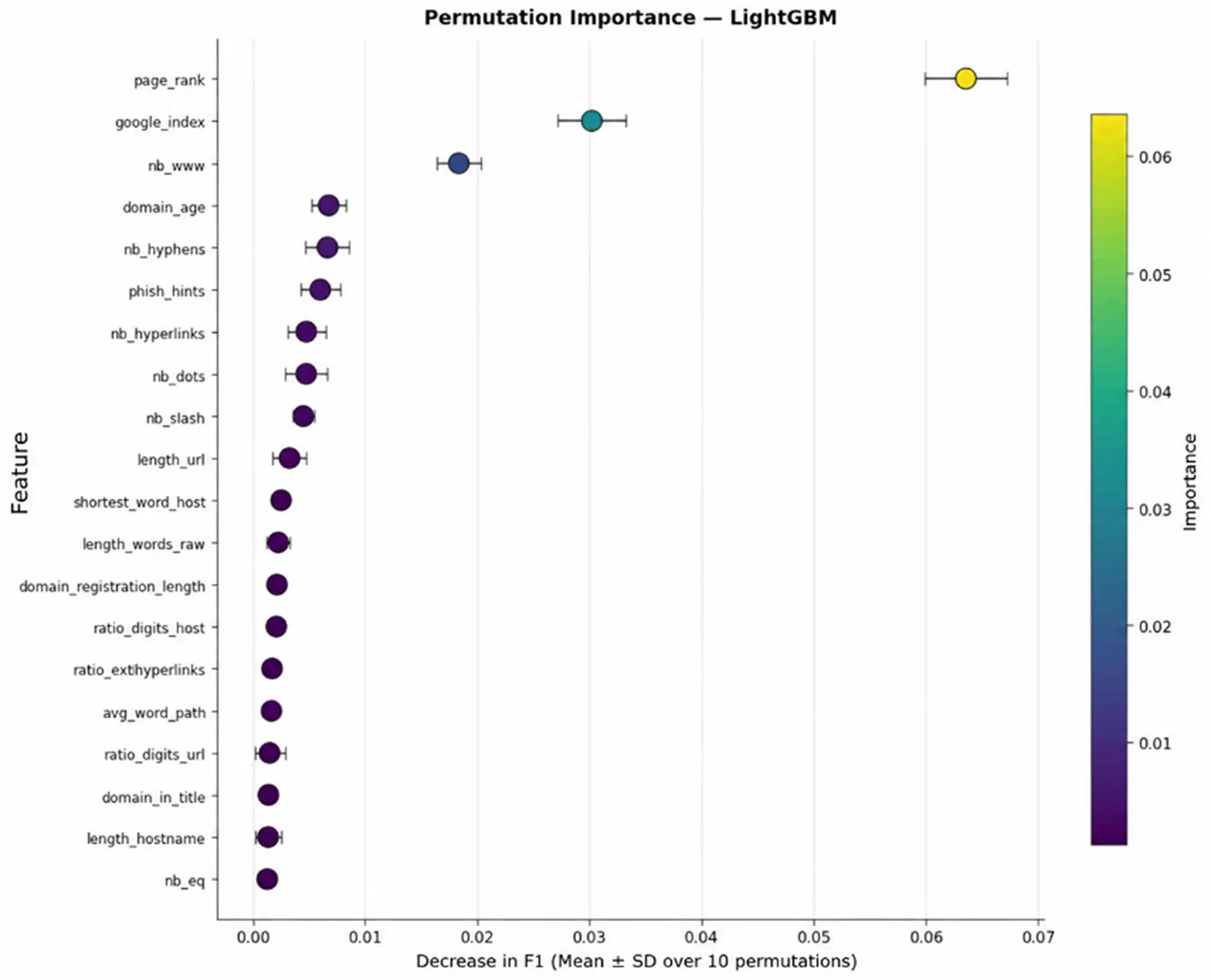
Permutation feature importance—LightGBM (decrease in *F*_1_, mean ± std over 10 permutations). Features are ranked by mean *F*_1_ decrease when their values are randomly shuffled in the test set. page_rank is the single most important feature under the permutation criterion (Δ*F*_1_≈0.065), followed by google_index (≈0.030) and nb_www (≈0.019). The color bar encodes importance magnitude (yellow = highest). Error bars represent the standard deviation across 10 independent permutations; the narrow bars for all top features confirm stable, reproducible importance estimates. The ordering is broadly consistent with the SHAP global ranking, providing cross-method validation of the feature relevance hierarchy.

**Figure 18:**
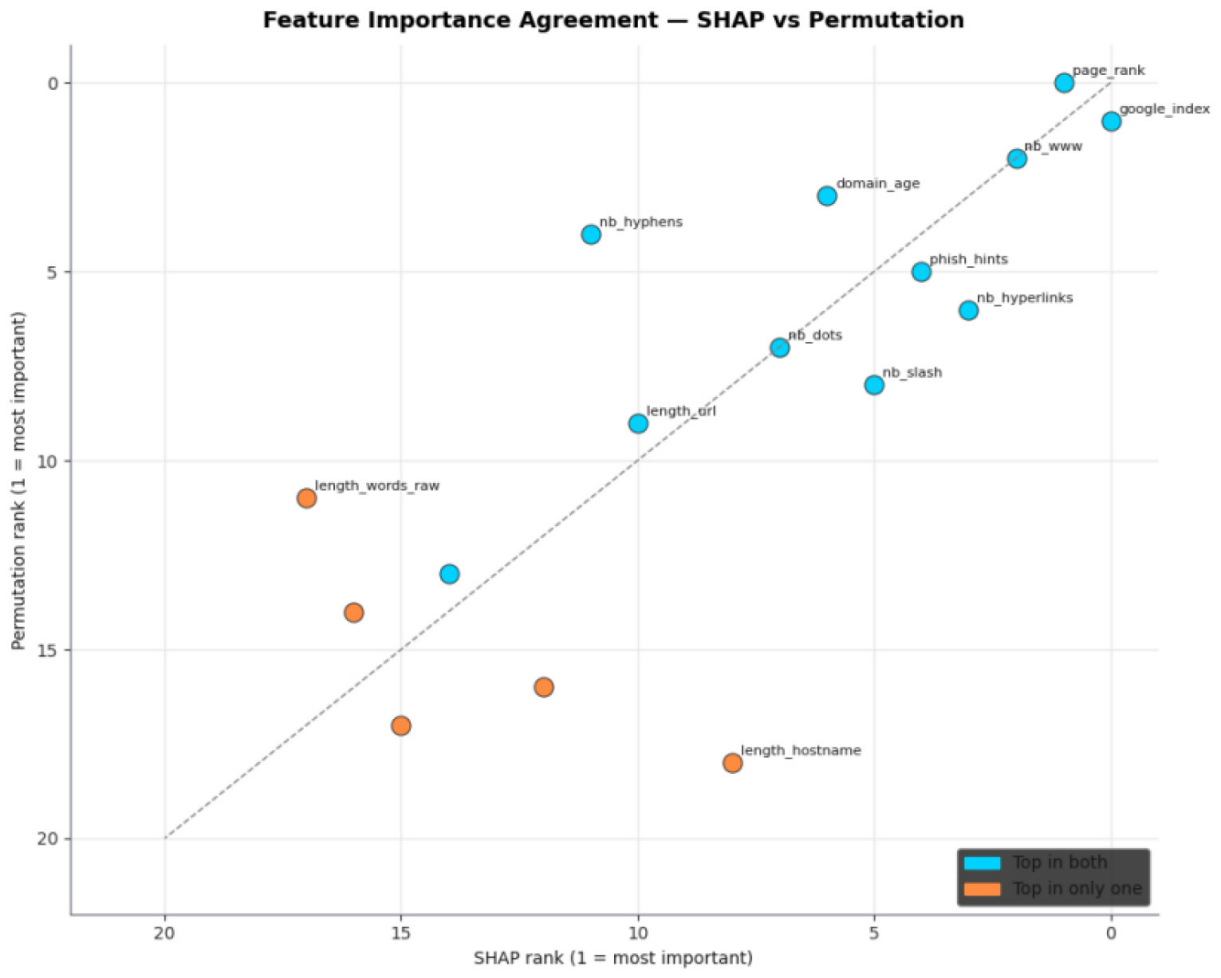
Feature importance agreement—SHAP vs. Permutation Importance (rank comparison). Each point represents one feature plotted by its SHAP rank (*x*-axis, lower = more important) against its Permutation Importance rank (*y*-axis, lower = more important); the dashed diagonal represents perfect rank agreement. Blue points (“Top in both”) are features appearing in the top-15 of both methods; orange points (“Top in only one”) appear in the top-15 of exactly one method. Eleven of 15 top features overlap (blue), demonstrating strong cross-method agreement (73% intersection). Features clustering near the diagonal (page_rank, google_index, nb_www, domain_age, phish_hints, nb_dots) are the most robustly identified across both attribution paradigms and constitute the irreducible core of the phishing detection signal.

**Table 7:** Top features under SHAP and Permutation Importance, with directional effect.

Rank	SHAP feature	Direction	Permutation feature
1	google_index	→ phishing	page_rank
2	page_rank	→ legitimate	google_index
3	nb_www	→ legitimate	nb_www
4	domain_age	→ legitimate	domain_age
5	phish_hints	→ phishing	nb_hyphens
6	nb_hyperlinks	→ legitimate	phish_hints
7	nb_dots	→ phishing	nb_dots
8	nb_hyphens	→ phishing	nb_hyperlinks
9	length_url	→ phishing	length_url

The intersection of the top-fifteen features under both methods contains 11 features, indicating strong agreement between the two attribution paradigms despite their orthogonal definitions, namely cooperative-game-theoretic vs. perturbation-based. The signed SHAP analysis exposes a coherent semantic narrative. Features whose elevation pushes a prediction toward phishing are predominantly URL-lexical signs of obfuscation: nb_dots, nb_hyphens, length_url, phish_hints, and the absence of nb_www, together with the negative reputation indicator google_index = 0. Features whose elevation pushes a prediction toward legitimacy are external-trust metrics: page_rank, domain_age, nb_hyperlinks, and web_traffic. This dichotomy provides a security-actionable rule of thumb: the more an URL relies on lexical complexity rather than reputational substance, the higher its phishing risk, and vice versa.

### 4.6 Ablation studies

Six ablation variants were constructed and evaluated to isolate the marginal contribution of each major design choice; the results are summarized in Table 8.

**Table 8:** Ablation study.

ID	Variant	*F*_1_	AUC	*ΔF*_1_
**LightGBM-side ablations (full:** *F*_1_ = 0.9699**, AUC** = 0.9943**)**				
A1	No standardization	0.9701	0.9942	+0.0002
A2	No feature selection (use all 87)	0.9682	0.9938	−0.0017
A3	Variance-threshold instead of MI	0.9614	0.9907	−0.0085
**Deep-network-side ablations (full:** *F*_1_ = 0.9652,				
**AUC** = **0.9903)**				
A4	BiLSTM only (drop CNN branch)	0.9657	0.9821	−0.0034
A5	CNN only (drop BiLSTM branch)	0.9663	0.9829	−0.0028
A6	No LightGBM-posterior augmentation	0.9496	0.9886	−0.0156

Each row removes or replaces one component of the full pipeline. Δ*F*_1_ is relative to the corresponding full model (LightGBM for A1–A3, CNN–BiLSTM for A4–A6).

Several conclusions follow. Standardization (A1) is essentially neutral for tree-based learners, as expected, since LightGBM is invariant to monotone feature transformations. Removing the mutual-information feature selection (A2) results in a small but consistent degradation, indicating that the 37 dropped features are noisy rather than informative under the LightGBM inductive bias. Replacing the mutual-information criterion with a variance-threshold criterion (A3) at the same retention budget reduces *F*_1_ by 0.0085, confirming that feature relevance to the target, rather than mere variability, is the appropriate selection signal. On the deep-network side, removing either the convolutional branch (A4) or the bidirectional LSTM branch (A5) reduces *F*_1_ by between 0.003 and 0.004, demonstrating that the two branches capture complementary patterns and that their concatenation produces a strict improvement over either alone. The most informative ablation is A6, which removes the LightGBM-posterior augmentation: *F*_1_ falls by 0.0156 to 0.9496, and AUC falls by 0.0017 to 0.9886. This is the largest single degradation observed in the entire study and provides the clearest evidence in our ablations for the proposed feature-extractor design. We stress that both the full hybrid and this ablation use the leakage-safe out-of-fold augmentation described in Section 3.9; the gain is therefore attributable to genuine predictive information transferred from the boosted-tree decision surface, not to in-sample posteriors.

### 4.7 Synthesis

The full experimental campaign supports four principal claims. First, gradient-boosted ensembles, especially LightGBM and XGBoost, are the most accurate single-stage classifiers on engineered phishing-URL features, with statistically equivalent performance to one another but significantly superior performance to all other model families. Second, preprocessing under strict train-only fitting eliminates leakage as an explanation for the high reported scores, indicating that the attainable performance of the engineered representation is intrinsically high rather than artifactual. Third, injecting tree-ensemble posterior probabilities into the input sequence of a CNN–BiLSTM hybrid yields a recall-favorable operating point useful in security-sensitive deployments without sacrificing overall accuracy, and the ablation analysis confirms that this augmentation is the dominant single contributor to the deep network's performance. Fourth, a small, semantically coherent subset of features anchored on Google indexing, page rank, domain age, and URL lexical complexity explains the bulk of the classifier's behavior and offers an operationally meaningful set of awareness indicators for end-user cybersecurity training.

## 5 Discussion

### 5.1 Implications for cybersecurity awareness

The convergent explanations produced by SHAP and Permutation Importance identify a small set of human-interpretable features whose values discriminate phishing from legitimate URLs with high reliability. These features sort cleanly into two groups, suggesting a compact pedagogical framing for end-user awareness training. The first group consists of external-reputation indicators that are typically opaque to end users but whose intuitive analogs, including “is this domain old and well-established?” and “does this domain appear in normal search results?”, are accessible through routine browser checks. The second group consists of URL-lexical complexity indicators that are directly visible in any address bar: unusually long URLs, large numbers of dots and hyphens, the absence of a recognizable second-level brand, and the presence of suspicious tokens.

To make this translation concrete rather than conceptual, we distill the convergent top-ranked features of Table 7 into the following compact set of candidate awareness guidelines, each annotated with the feature(s) from which it derives. The guidelines are intended to be memorable heuristics for end users, not detection rules for automated systems.

1. **Read the whole address before the first single slash**. The genuine destination is the registered domain immediately to the left of the first lone “/”; brand names appearing only in the path or in subdomains are not the destination. *(derives from* nb_dots, nb_www*, absence of a second-level brand)*
2. **Treat unusually long or punctuation-heavy URLs as a warning**. Addresses that are very long or crowded with dots and hyphens are disproportionately phishing. *(derives from* length_url, nb_dots, nb_hyphens*)*
3. **Be suspicious of alarming or action-urging words inside the link itself**. Tokens such as secure, account, verify, login, and update embedded in the URL string are common phishing hints. *(derives from* phish_hints*)*
4. **Expect a real brand to have a search-visible, established domain**. If a link claims to be a well-known organization but the domain does not appear in ordinary search results, this mismatch is among the strongest single indicators of phishing. *(derives from* google_index, page_rank*)*
5. **Prefer domains with a visible history**. Very recently registered domains, and domains registered for only a short period, are markedly more likely to be malicious; when in doubt, a quick public WHOIS or domain-age check is informative. *(derives from* domain_age, domain_registration_length*)*
6. **Match surface complexity against reputational substance**. The unifying rule: when the surface form of a URL is complex, the underlying domain should command commensurate reputational evidence; a complex address on an obscure, new, or unindexed domain should be treated as suspect. *(synthesizes both feature groups)*

The dichotomy between trust-by-reputation and risk-by-lexical-complexity underlies all six guidelines and yields a teachable principle that does not require users to become security experts. This framing is consistent with empirical findings on the effectiveness of brief, principle-based cybersecurity training over rote memorization of indicators of compromise (Abawajy, 2014; Jampen et al., 2020).

Two important caveats bound the strength of these guidelines. First, they are derived from the global feature attributions of the LightGBM model rather than from the deployed CNN–BiLSTM hybrid; while the convergent SHAP and Permutation rankings provide strong evidence for the underlying feature importance, the assumption that the same hierarchy governs the hybrid's behavior is not directly validated here, and Section 5 identifies gradient-based attribution of the hybrid as necessary future work. The guidelines should therefore be read as LightGBM-derived heuristics whose transfer to the hybrid is plausible but unverified. Second, and more fundamentally, these are guidelines inferred from model behavior, not behavioral evidence: We conducted no user study, and the claim that they improve human phishing-detection performance remains a hypothesis. We accordingly present them as candidate guidelines that *may* inform awareness strategies, and we flag a controlled user study as the appropriate next step to establish their pedagogical value.

### 5.2 Comparison with the literature

The performance achieved on the corpus considered is comparable to the upper end of the published range for engineered-feature phishing detection (Alsariera et al., 2020; Gandotra and Gupta, 2021; Hannousse and Yahiouche, 2021). The methodological contributions of the present study, in particular the explicit leakage controls, the Wilcoxon-based significance testing, the dual-method explanation analysis, and the ablation protocol, are intended to make this performance level reproducible and interpretable rather than merely reportable. The finding that LightGBM and XGBoost are statistically indistinguishable on this corpus is consistent with several recent comparative studies in adjacent cybersecurity domains (Shwartz-Ziv and Armon, 2022) and suggests that future benchmarks should report not only point performance but also fold-level distributions to enable principled selection.

### 5.3 Limitations

Three limitations should be acknowledged. First, the corpus, while large by historical standards, represents a single public dataset captured at one point in time, and the distribution of phishing URLs is known to drift as adversaries adapt to deployed detectors (Tian et al., 2018). The present evaluation includes no temporal split, no cross-dataset transfer test, and no external validation corpus; consequently, the performance levels reported here should be interpreted as an upper bound on the steady-state operating performance of a deployed system rather than as evidence of cross-distribution generalization. Establishing the latter requires (i) a chronological train-on-past, test-on-future split to quantify concept-drift decay, and (ii) evaluation on at least one independently collected phishing corpus to measure cross-dataset transfer; we identify both as essential directions for future work rather than claims of the present study. Continual retraining on fresh data is in any case essential for sustained deployment. Second, the feature representation depends on external services for WHOIS lookups, page-rank queries, and traffic estimates, which introduce latency and a soft attack surface, since adversaries may attempt to manipulate these signals (Moghimi and Varjani, 2016). Third, the explainability analysis is conducted at the level of the LightGBM model rather than the deployed CNN–BiLSTM hybrid; while the convergent SHAP and Permutation rankings provide strong evidence for the underlying feature importance, future work should extend the analysis to the deep-network branch through gradient-based attribution methods such as Integrated Gradients (Sundararajan et al., 2017).

### 5.4 Future directions

Several extensions of the present framework are immediate. The feature-extractor construction generalizes beyond LightGBM, and an ensemble of feature extractors drawn from the top-three classifiers could provide a more robust embedding. The CNN–BiLSTM architecture could be augmented with a self-attention layer over the bidirectional hidden states (Vaswani et al., 2017) to make long-range dependencies between features more salient. On the awareness side, the convergent feature rankings could be operationalised as a browser extension that exposes the dominant indicators to users in real time, providing a passive training signal during routine browsing. Finally, the performance evaluation should be replicated on temporally split corpora to quantify the rate of concept drift and the corresponding retraining cadence required to maintain operational performance.

## 6 Conclusion

This study presented a complete machine learning framework for phishing-URL detection over a balanced corpus of 11,430 instances and 87 engineered features. Eleven heterogeneous classifiers were trained under a strict no-leakage protocol, and the Light Gradient Boosting Machine emerged as the strongest single-stage model with *F*_1_ = 0.9699 and AUC = 0.9943. Wilcoxon signed-rank testing on 10-fold *F*_1_-scores confirmed that LightGBM significantly outperforms every other classifier evaluated at this stage (*p* ≤ 0.002) except XGBoost, with which it is statistically equivalent (*p* = 0.375); three of the slowest underperforming models were excluded from this stage on computational grounds and are therefore outside the scope of this significance claim. A hybrid CNN–BiLSTM architecture, augmented with the out-of-fold posterior probabilities of the LightGBM extractor, achieved *F*_1_ = 0.9652 and recall = 0.9703. The hybrid does not improve aggregate *F*_1_ over LightGBM but provides a recall-favorable operating point of practical value in deployments where missed detections are costlier than false alarms. Ablation studies confirmed the contribution of every major design choice, with the LightGBM-posterior augmentation contributing the largest single performance gain to the deep network. Tree SHAP and Permutation Importance produced convergent global rankings, with 11 of 15 top features overlapping, and identified external-reputation indicators (Google indexing, page rank, domain age, and web traffic) and URL-lexical complexity indicators (length, dots, hyphens, and phishing hints) as the two semantically coherent axes governing classifier behavior. These convergent findings are used to motivate a compact set of candidate awareness guidelines that may inform end-user cybersecurity training, connecting technical detection to the human factors that ultimately shape the security posture of digital infrastructure.

## Data Availability

The original contributions presented in the study are included in the article/supplementary material, further inquiries can be directed to the corresponding author.
